# H3K36me2 methyltransferase NSD2/WHSC1 promotes triple-negative breast cancer metastasis via activation of ULK1-dependent autophagy

**DOI:** 10.1080/15548627.2025.2479995

**Published:** 2025-03-25

**Authors:** Danyang Chen, Xiaohui Chen, Mingqiang Yang, Qiunuo Li, Shaojuan Weng, Siyue Kou, Xi Liu, Guanmin Jiang, Hao Liu

**Affiliations:** a Guangzhou Institute of Cancer Research, The Affiliated Cancer Hospital, Guangzhou Medical University, Guangzhou, Guangdong, China; b Department of Clinical Laboratory, The Fifth Affiliated Hospital, Sun Yat-sen University, Zhuhai, Guangdong, China; c The Molecular Diagnosis Center, Yunnan Cancer Hospital, The Third Affiliated Hospital of Kunming Medical University Peking University Cancer Hospital, Kunming, Yunnan, China

**Keywords:** Autophagy, H3K36me2, metastasis, NSD2, triple-negative breast cancer, ULK1

## Abstract

Metastasis is the primary cause for treatment failure and poor prognosis in patients with triple-negative breast cancer (TNBC). Macroautophagy/autophagy plays a crucial role in tumor growth and metastasis. Genetic or epigenetic regulation of autophagy-related factors alters autophagy levels, which subsequently promotes cancer progression and affects the therapeutic effectiveness. However, the molecular basis for the transcriptional and epigenetic regulation of autophagy in TNBC progression is poorly understood. In this study, we reveal the histone methyltransferase NSD2/WHSC1 (nuclear receptor binding SET domain protein 2) as a novel epigenetic regulator of autophagy in TNBC progression. We demonstrate that the expression of NSD2 is significantly upregulated in TNBC cells and high NSD2 expression is correlated with poor TNBC survival. Elevated expression of NSD2 significantly promotes TNBC metastasis in multiple TNBC models. Mechanistically, ULK1 (unc-51 like autophagy activating kinase 1) is identified as a novel target of NSD2 and NSD2-mediated histone H3K36me2 methylation directly activates ULK1 transcription in TNBC cells. Notably, NSD2-induced ULK1 expression facilitates autophagosome maturation and increases autophagic flux, thus promoting autophagy-related malignancy progression in TNBC. Furthermore, pharmacological inhibition of NSD2 using MS159 and MCTP-39 significantly suppresses TNBC autophagy, growth, and metastasis both *in vivo* and *in vitro*. In conclusion, our findings demonstrate a pivotal epigenetic role for the NSD2-H3K36me2 axis in regulating ULK1 expression and identify a novel NSD2-ULK1-autophagy signaling axis in the promotion of TNBC progression, suggesting that NSD2 inhibition may be an effective treatment strategy for TNBC.

**Abbreviations**: CDH2/N-cadherin: cadherin 2; ChIP: chromatin immunoprecipitation; EMT: epithelial-mesenchymal transition; ESR: estrogen receptor; FN1: fibronectin 1; GEPIA: Gene Expression Profiling Interactive Analysis; H3K36me2: di-methylation at lysine 36 of histone 3; H&E: hematoxylin and eosin; HDM: histone demethylase; HMT: histone methyltransferase; HIF1A/HIF-1α: hypoxia inducible factor 1 subunit alpha; IF: Immunofluorescence; IHC: Immunohistochemistry; NSD: nuclear receptor binding SET domain protein; PGR: progesterone receptor; qRT-PCR: quantitative RT-PCR; TCGA: The Cancer Genome Atlas; TNBC: triple-negative breast cancer; TSS: transcription start site; ULK1: unc-51 like autophagy activating kinase 1.

## Introduction

Breast cancer is the most commonly diagnosed cancer type and the leading cause of cancer-related death in women worldwide [1]. Triple-negative breast cancer (TNBC) is characterized by the lack of ESR (estrogen receptor) and PGR (progesterone receptor) expression and the absence of ERBB2/HER2 (erb-b2 receptor tyrosine kinase 2) overexpression or gene amplification [2]. TNBC accounts for 15–20% of all breast cancer cases and is a biologically aggressive tumor with higher incidence of distant metastasis and worse prognosis. Approximately 46% of TNBC patients will experience distant metastasis in the 3^rd^ year after diagnosis. The median survival time after metastasis is only 13.3 months under the current treatment [3]. However, therapies directed to specific molecular targets have rarely achieved clinically meaningful improvements in the survival of patients with metastatic TNBC [2,4]. Therefore, understanding precise mechanisms underlying TNBC metastasis is imperative and effective treatments are emerging.

Autophagy is a highly conserved catabolic process that degrades unfolded proteins and damages organelles by formation of autophagosomes, which is responsible for maintaining cellular function and homeostasis [5]. Several studies have shown that autophagy is inextricably linked with TNBC progression [6]. Considering its aggressive nature, TNBC exhibits a higher level of autophagy than other breast cancer subtypes [7]. Expression of the autophagy-related proteins, such as MAP1LC3A/LC3A, and LC3B, are higher in TNBC than other subtypes [8]. In addition, recent studies have reported that autophagy is involved in cancer progression, including invasion and metastasis [7,9]. Autophagy promotes tumor invasion and metastasis through enhancing cancer stem cell phenotype, regulating cell adhesion, inducing epithelial-mesenchymal transition (EMT), and providing the energy for tumor cell invasion [9]. Therefore, blocking autophagy has been suggested as a promising approach for TNBC treatment.

Accumulating evidence indicated that dysregulation of epigenetic modifications, including histone modifications, DNA methylation, chromatin remodeling, and noncoding RNA expression, are closely associated with the development and progression of various malignancies [10]. Recent evidence have shed light on the influence of histone modifications underlying tumor development from tumor initiation to distant metastasis in TNBC [11]. An essential histone modification involved in TNBC progression is histone methylation, which is regulated by histone methyltransferases (HMTs) and histone demethylases (HDMs) that add and remove methyl groups, respectively, on lysine and arginine residues within histone H3 and H4 [12]. Aberrant histone methylation and demethylation play diverse roles in TNBC progression and account for lower survival in patients with TNBC [13].

NSD2/WHSC1/MMSET (nuclear receptor binding SET domain protein 2) is a SET domain-containing histone methyltransferase that catalyzes di-methylation at lysine 36 of histone H3 (H3K36me2) [14–16]. Widespread distribution of H3K36me2 is associated with active transcription of a series of genes [17–19]. The function of NSD2 is critical for early mammalian development [20]. In recent years, NSD2 expression is found to be overexpressed in multiple myeloma, colon cancer, prostate cancer, and lung cancer [21]. Increasing evidence has implicated NSD2 in the pathogenesis of diverse solid tumors [16,22–25]. For example, NSD2 promotes tumor angiogenesis through methylating and activating STAT3 protein in colon cancer [26]. Li N *et al*. reported that NSD2 enhances cancer metastasis by targeting MTORC2 signaling in prostate cancer [27]. However, the biological function and potential mechanism of NSD2 in TNBC progression remain unclear.

Herein, we demonstrate that NSD2 expression is significantly upregulated in TNBC cells and tissues. NSD2-mediated H3K36me2 activates ULK1 transcription, driving autophagy to accelerate TNBC progression both *in vitro* and *in vivo*. Pharmacological inhibition of NSD2 significantly suppresses autophagy, growth, and metastasis in TNBC. Our findings suggest that targeting NSD2 May be a promising therapeutic strategy for patients with TNBC.

## Results

### NSD2 is upregulated in TNBC and associated with poor survival

**Figure 1.**
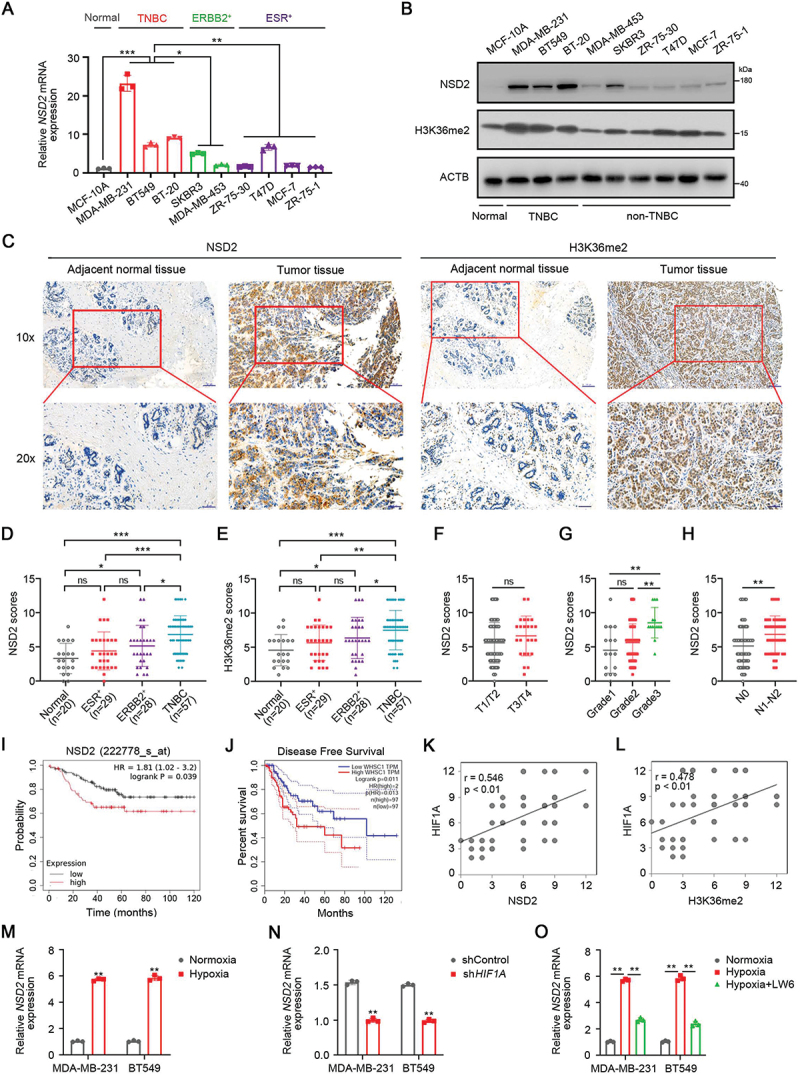
NSD2 is upregulated in TNBC and is associated with poor survival. (A and B) The expression of *NSD2* in a panel of 9 breast cancer lines and normal MCF-10A cells were measured by qRT-PCR (A) and by western blotting (B). (C) Representative images of NSD2 and H3K36me2 staining by IHC analysis in breast cancer specimens. The breast cancer tissue sections were quantitatively scored according to the percentage of positive cells and staining intensity. Scale bar: 10×, 100 μm; 20×, 50 μm. (D) IHC staining scores for NSD2 in adjacent normal tissues and breast cancer tissues. (E) IHC staining scores for H3K36me2 in adjacent normal tissues and breast cancer tissues. (F) Correlation of NSD2 expression with the T stage of breast cancer tissues. (G) Correlation of NSD2 expression with the histological grade of breast cancer tissues. (H) Correlation of NSD2 expression with the lymph node metastasis of breast cancer tissues. (I and J) Survival curves of TNBC patients with low expression versus high expression of NSD2 in TCGA database (I) and GEPIA database (J). (K) Correlation between protein expression of HIF1A and NSD2. (L) Correlation between protein expression of HIF1A and H3K36me2. (M) TNBC cells were cultured under normoxia (20% O_2_) or hypoxia (1% O_2_), and expression of *NSD2* was detected using qRT-pcr. (N) TNBC cells were transfected with *HIF1A* shRNA or control shRNA, the expression of *NSD2* was detected using qRT-pcr. (O) TNBC cells following LW6 treatment (10 μM) or vehicle were cultured under normoxia (20% O_2_) or hypoxia (1% O_2_), and expression of *NSD2* was detected using qRT-pcr. Error bars represented the mean ± sem and the dots represented the value of each experiment. ns, no significance, *p < 0.05, **p < 0.01, ***p < 0.001.

To explore the roles and mechanisms of NSD2 in TNBC progression, we first examined NSD2 expression in a panel of 9 commonly studied breast cancer lines that correlated with the receptor status. Our results showed that TNBC cell lines had substantially elevated *NSD2* mRNA and protein expression compared to that in normal MCF-10A cells and receptor-positive cell lines (Figure 1A,B, Figure S1A). Consistently, the protein expression of H3K36me2 was increased in TNBC cells (Figure 1B, Figure S1A). We further examined NSD2 and H3K36me2 expression in 114 independent primary breast tumor tissues and 20 adjacent normal tissues by immunohistochemistry analysis. The results showed that NSD2 and H3K36me2 were significantly upregulated in TNBC tissues compared to adjacent normal tissues (Figure 1C-E). More importantly, 57 TNBC tumor tissues expressed significantly elevated NSD2 and H3K36me2 protein levels compared to the receptor-positive tumors (Figure 1D,E). We further investigated possible correlation between NSD2 expression and clinicopathological factors. No association was observed between NSD2 and the T stage (*p* = 0.2319) (Figure 1F). However, patients with high NSD2 expression demonstrated a high histological grade (Figure 1G), and increased lymph node metastasis (Figure 1H). Furthermore, the correlation between NSD2 expression and overall survival of the TNBC patients was investigated. TCGA analysis results showed that TNBC patients with high *NSD2* expression had poorer overall survival than those with low *NSD2* expression (Figure 1I,J), suggesting that NSD2 upregulation predicts poor prognosis in patients with TNBC.

Furthermore, correlations between expression of *HIF1A/HIF-1α* and *NSD2* were determined via GEPIA database and TCGA database, revealing that *NSD2* was positive correlated with *HIF1A* (Figure S1B). We then investigated the expression levels of the HIF1A in breast tumor tissues. We found that the expression of HIF1A was markedly increased in TNBC tumors compared to the adjacent normal tissues (Figure S1C). We further observed a positive correlation between HIF1A and NSD2 or H3K36me2 (Figure 1K,L), providing evidence for hypoxia-mediated NSD2 upregulation. Then, qRT-PCR and western blotting were used to investigate the effect of hypoxia on NSD2 expression. The results showed that *NSD2* mRNA and protein levels were increased in TNBC cells under hypoxic conditions (Figure 1M, Figure S1D-E). However, NSD2 expression in normal MCF-10A cells and ESR-positive MCF-7 cells showed no obvious change under hypoxic conditions (Figure S1F-G). Moreover, we found that NSD2 expression was decreased in *HIF1A*-knockdown TNBC cells (Figure 1N, Figure S1H-I). Consistently, the expression of NSD2 was decreased in TNBC cells under hypoxia after treatment with selectively HIF1A inhibitor LW6 (Figure 1O, Figure S1J-K), suggesting that HIF1A is responsible for NSD2 upregulation in TNBC cells. Taken together, these results indicate that *NSD2* is a novel target gene of HIF1A, and its upregulation is associated with poor survival of patients with TNBC.

### NSD2 promotes TNBC invasion and metastasis both in vitro and in vivo

**Figure 2.**
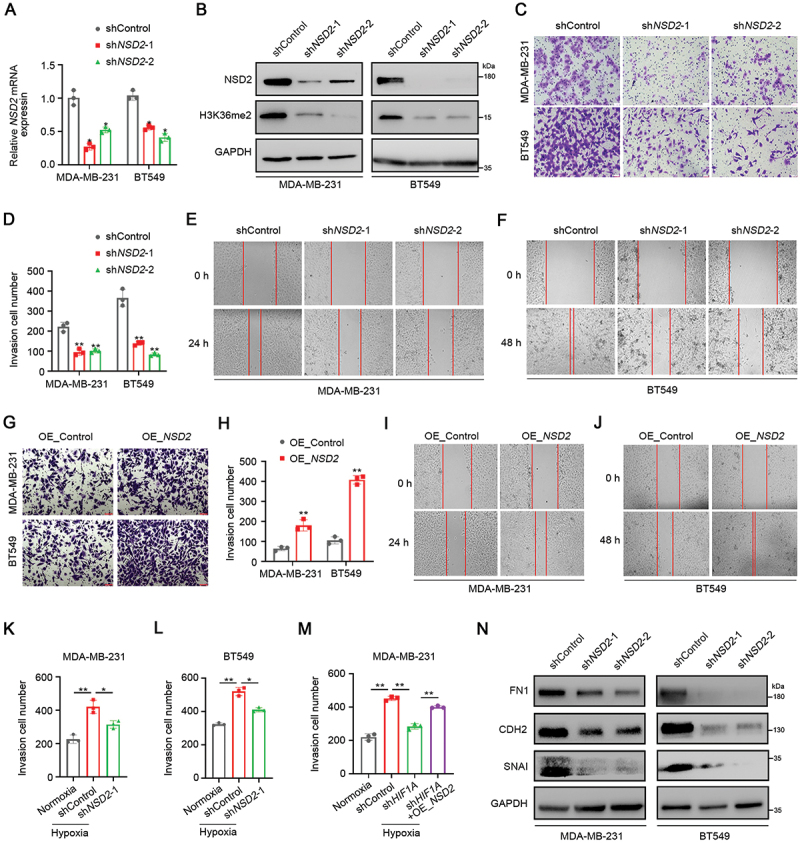
NSD2 promotes TNBC migration and invasion *in vitro*. (A) MDA-MB-231 and BT549 cells were stably transfected with *NSD2* shRNA or control shRNA, the expression of *NSD2* was measured by qRT-pcr. (B) MDA-MB-231 and BT549 cells were stably transfected with *NSD2* shRNA or control shRNA, the expression of NSD2 and H3K36me2 were measured by western blotting. (C and D) MDA-MB-231 and BT549 cells were stably transfected with *NSD2* shRNA or control shRNA, cell invasion ability was measured by transwell assay. Scale bar: 100 μm. (E and F) MDA-MB-231 and BT549 cells were stably transfected with *NSD2* shRNA or control shRNA, cell migration ability was measured by wound healing assay. Scale bar: 100 μm. (G and H) MDA-MB-231 and BT549 cells were stably transfected with *NSD2*-overexpressing vector, cell invasion ability was measured by transwell assay. Scale bar: 100 μm. (I and J) MDA-MB-231 and BT549 cells were stably transfected with *NSD2*-overexpressing vector, cell migration ability was measured by wound healing assay. Scale bar: 100 μm. (K and L) Cell invasion ability of *NSD2*-knockdown MDA-MB-231 and BT549 cells under normoxia and hypoxia was measured by transwell assay. Scale bar: 100 μm. (M) MDA-MB-231 cells co-transfected with *HIF1A* shRNA and *NSD2*-overexpressing vector, cell invasion ability was measured by transwell assay under normoxia and hypoxia. (N) MDA-MB-231 and BT549 cells were stably transfected with *NSD2* shRNA or control shRNA, the expression levels of emt-related factors FN1, CDH2, and SNAI were detected by western blotting. Error bars represented the mean ± sem and the dots represented the value of each experiment. *p < 0.05, **p < 0.01.

Given that NSD2 is upregulated in TNBC tissues and is associated with metastasis, we then investigated whether NSD2 influences TNBC cell metastatic capacity. Two *NSD2* targeted shRNAs (sh*NSD2*) were used to knockdown *NSD2* expression in TNBC cells (Figure 2A,B, Figure S2A). Accordingly, knockdown of *NSD2* significantly decreased the levels of H3K36me2 (Figure 2B, Figure S2A). Transwell assays and wound healing assays showed that knockdown of *NSD2* in MDA-MB-231 and BT549 cells significantly inhibited cell invasion and migration compared to that in the controls, respectively (Figure 2C-F). Conversely, ectopic expression of NSD2 in MDA-MB-231 and BT549 cells (Figure S2B-D) markedly increased their invasion and migration ability (Figure 2G-J). Moreover, we found that re-expression of *NSD2* in *NSD2*-knockdown BT549 cells effectively rescued cell invasive abilities (Figure S2E-F). We next analyzed the role of NSD2 in hypoxia-induced TNBC cell invasion. The promoting effect of hypoxia on cell invasion was markedly reversed by *NSD2* knockdown (Figure 2K,L, Figure S2G). Moreover, we found that hypoxia-induced cell invasion was impaired in *HIF1A*-knockdown MDA-MB-231 cells, while it was enhanced in sh*HIF1A* + OE_*NSD2* cells compared to sh*HIF1A* + OE_Control cells (Figure 2M, Figure S2H), suggesting that NSD2 acts as an effector of HIF1A in hypoxia-induced cell invasion of TNBC cells. EMT is essential for the invasion and metastatic potential of tumor cells [28]. We then examined the effect of NSD2 on EMT and found that the knockdown of *NSD2* significantly decreased the expression of mesenchymal marker FN1 (fibronectin 1) and CDH2/N-cadherin as well as EMT transcriptional factor SNAI/Snail (Figure 2N, Figure S2I). Nevertheless, we observed an increased FN1, CDH2, and SNAI expression in *NSD2*-overexpressing MDA-MB-231 and BT549 cells (Figure S2C-D). These results suggest that NSD2 enhances TNBC cell invasion and migration via EMT.

**Figure 3.**
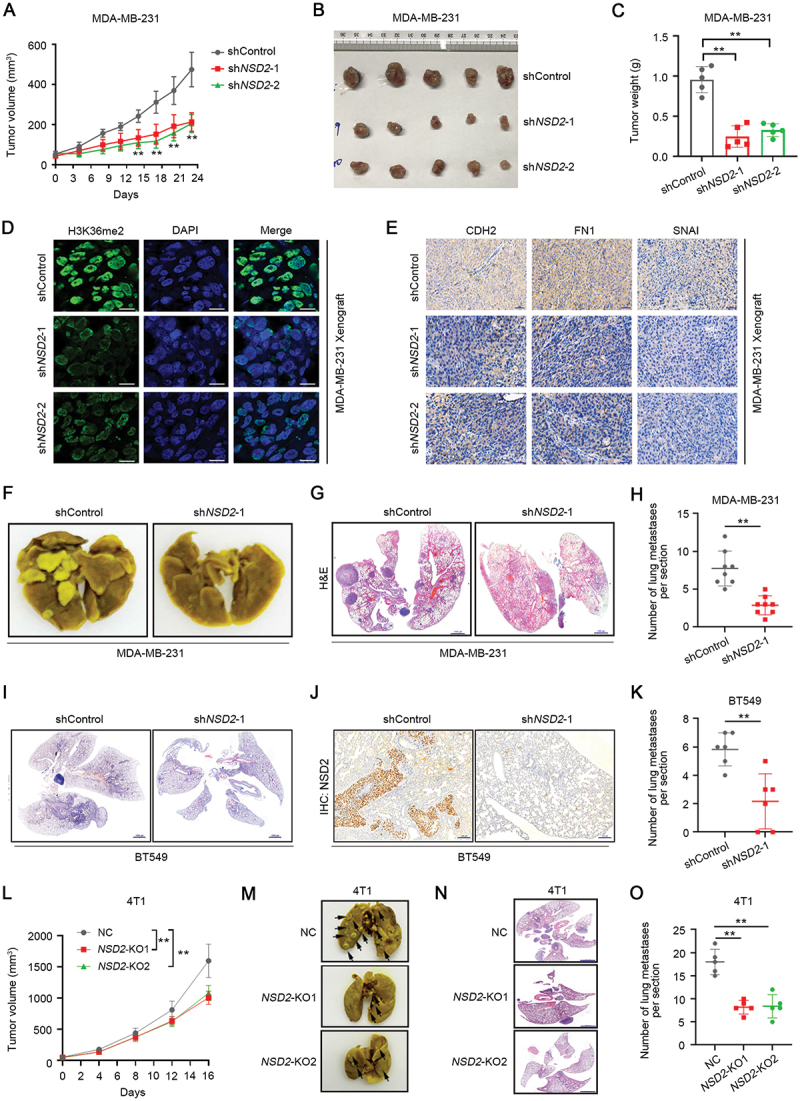
NSD2 promotes TNBC metastasis *in vivo*. (A-C) A total of 5 × 10^6^ MDA-MB-231/*NSD2* shRNA or MDA-MB-231/Control shRNA cells were inoculated subcutaneously into the female nude mice (*N* = 5 per group). Tumor size was measured at indicated time intervals, tumor volume was calculated and growth curves were plotted using average tumor volume within each experimental group at the set time points (A). At the end of treatment, tumors were excised and imaged (B). Tumor weights were measured (C). (D) Tumor tissues were fixed, sectioned, and placed on slides. Tumor specimens were subjected to immunofluorescence staining for H3K36me2. Scale bar: 50 μm. (E) Tumor specimens were subjected to IHC staining for FN1, CDH2, and SNAI. Scale bar: 100 μm. (F-H) A total of 1 × 10^6^ MDA-MB-231/*NSD2* shRNA or MDA-MB-231/Control shRNA cells were tail vein-injected into nude mice (*N* = 8 per group), representative images showing lung nodules (F). Representative H&E images of lung tissues showing metastatic nodules, scale bar: 1000 μm (G). Average number of lung metastases nodules in the indicated groups (H). (I-K) A total of 1 × 10^6^ BT549/*NSD2* shRNA or BT549/Control shRNA cells were tail vein-injected into nude mice (*N* = 6 per group), representative H&E images of lung tissues showing metastatic nodules, scale bar: 1000 μm (I). Representative IHC staining for NSD2 in lung sections, scale bar: 200 μm (J). Average number of lung metastases nodules of indicated groups (K). (L-O) A total of 1 × 10^5^ 4T1/*NSD2* KO or 4T1/NC cells were injected into the mammary fat pads of female nude mice (*N* = 5 per group). Tumor size was measured at indicated time intervals. Tumor volume was calculated and growth curves were plotted using average tumor volume within each experimental group at the set time points (L). Representative images showing lung nodules (M). Representative H&E images of lung tissues showing metastatic nodules, scale bar: 1000 μm (N). Average number of lung metastases nodules in the indicated groups (O). Error bars represented the mean ± sem and the dots represented the value of each experiment. *p < 0.05, **p < 0.01.

To further validate that NSD2 promotes TNBC progression *in vivo*, we subcutaneously injected TNBC cells into nude mice. Knockdown of *NSD2* could markedly inhibit tumor growth of MDA-MB-231 cells, as evidenced by a significant reduction of tumor size and weight when compared to the non-target shRNA control (Figure 3A-C). Similarly, we also observed a significant decrease in *NSD2*-knockdown BT549 tumor growth in the mice as compared to control (Figure S3A-C). Immunofluorescence staining showed that the expression of H3K36me2 was significantly decreased in the tumors from the *NSD2*-knockdown groups (Figure 3D, Figure S3D). Moreover, consistent with our *in vitro* results, the expression of FN1, CDH2, and SNAI were significantly decreased in the tumors from the *NSD2*-knockdown groups (Figure 3E, Figure S3E). In the experimental metastasis assay, nude mice tail-vein injected with *NSD2*-knockdown MDA-MB-231 cells displayed fewer lung metastatic colonies (Figure 3F-H). Similarly, we also observed a significant decrease in lung metastatic colonies in the mice tail-vein injected with *NSD2*-knockdown BT549 cells as compared to control cells (Figure 3I-K). Moreover, orthotropic breast cancer model was established by planting the *NSD2*-knockout 4T1 cells (4T1/*NSD2*-KO) or control cells (Figure S3F-G) into the mammary fat pads of BALB/c nude mice. We observed that knockout of *NSD2* significantly decreased 4T1 orthotopic tumor growth (Figure 3L). In vivo imaging assay and HE staining assay showed that knockout of *NSD2* significantly inhibited the lung metastasis of 4T1 orthotopic tumors, as reflected by the reductions in the numbers of lung metastatic lesions (Figure 3M-O). Taken together, these results support a critical role of NSD2 in promoting TNBC metastasis.

### NSD2-mediated H3K36me2 promotes ULK1 expression in TNBC

**Figure 4.**
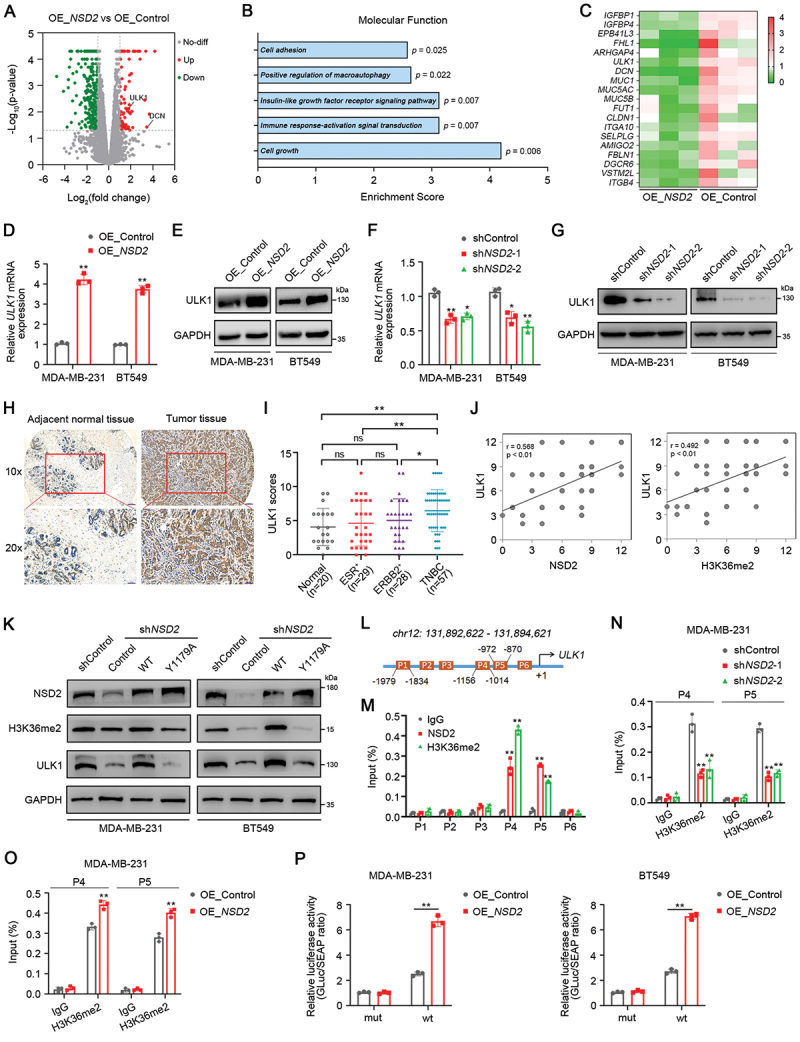
NSD2-mediated H3K36me2 promotes ULK1 expression in TNBC. (A) Volcano plots showing the differentially expressed genes in MDA-MB-231 cells stably transfected with control vector (OE_control) or *NSD2*-overexpressing vector (OE_*NSD2*) by rna-seq. Red dots represented genes with a log2 (Fold change) > 2 and false discovery rate (FDR) < 0.05. (B) Gene ontology (GO) enrichment analysis on upregulated genes representing distinct molecular functions enriched in *NSD2*-overexpressing MDA-MB-231 cells versus control cells. (C) Heat map showing the indicated genes of enriched molecular functions. (D and E) MDA-MB-231 or BT549 cells were stably transfected with *NSD2*-overexpressing vector or control vector, the expression levels of *ULK1* were analyzed by qRT-pcr (D) and western blotting (E). (F and G) MDA-MB-231 or BT549 cells were stably transfected with *NSD2* shRNA or control shRNA, the expression levels of *ULK1* were analyzed by qRT-pcr (F) and western blotting (G). (H) Representative images of ULK1 staining by IHC analysis in breast cancer specimens. Scale bar: 10×, 100 μm; 20×, 50 μm. (I) IHC staining scores for NSD2 in adjacent normal tissues and breast cancer tissues. (J) Correlation between protein expression of ULK1 and NSD2 or H3K36me2, respectively. (K) Wild-type *NSD2* or *NSD2*^*Y1179A*^ mutant vector was transfected in *NSD2*-knockdown MDA-MB-231 and BT549 cells, the expression of NSD2, H3K36me2, and ULK1 were analyzed by western blotting. (L) Schematic diagram of the primer pair location in the *ULK1* promoter. (M) ChIP analyses of the *ULK1* promoter in MDA-MB-231 cells by use of antibodies against NSD2 and H3K36me2. (N) ChIP analyses of the *ULK1* promoter in MDA-MB-231 cells transfected with *NSD2* shRNA or control shRNA using the antibody against H3K36me2. (O) ChIP analyses of the *ULK1* promoter in MDA-MB-231 cells transfected with *NSD2*-overexpressing vector or control vector using the antibody against H3K36me2. (P) MDA-MB-231 and BT549 cells were transfected with *NSD2*-overexpressing vector or control vector, followed by transfection with a luciferase reporter construct containing the wild-type or mutant *ULK1* promoter, the relative luciferase activities were analyzed. Error bars represented the mean ± sem and the dots represented the value of each experiment. ns, no significance, *p < 0.05, **p < 0.01.

To elucidate the mechanisms underlying the tumor-promoting roles of NSD2, we analyzed the differentially expressed genes after *NSD2* overexpression using RNA sequencing. RNA sequencing revealed that 73 transcripts were significantly upregulated, while 278 transcripts were significantly downregulated in *NSD2*- overexpressing MDA-MB-231 cells (Fold change > 2 and *p* < 0.05) (Figure 4A). Given that NSD2-mediated H3K36me2 is associated with active gene transcription, we focused on the 73 upregulated transcripts. Gene ontology (GO) enrichment analysis showed that upregulated genes representing distinct biological pathways were significantly enriched, including cell growth (*EPB41L3*, *IGFBP4*, *IGFBP1*, *FHL1*, *ARHGAP4*, *ULK1*), immune response-activating signal transduction (*MUC1*, *MUC5AC*, *MUC5B*), insulin-like growth factor receptor signaling pathway (*IGFBP1*, *IGFBP4*), positive regulation of macroautophagy (*ULK1*, *DCN*), and cell adhesion (*FUT1*, *VSTM2L*, *AMIGO2*, *ITGB4*, *ITGA10*, *FBLN1*, *CLDN1*, *MUC1*, *SELPLG*, *DGCR6*) (Figure 4B,C). Intriguingly, the mRNA expression of *ULK1* (unc-51 like autophagy activating kinase 1), one of the core human autophagy-related genes [29], was significantly increased in *NSD2*-overexpressing MDA-MB-231 cells as compared to that in control cells (Figure 4C, Figure S4A). qRT-PCR and western blotting further confirmed that overexpression of *NSD2* significantly increased the expression of ULK1 (Figure 4D,E, Figure S4B). In contrast, knockdown of *NSD2* decreased the expression of ULK1 in TNBC cells (Figure 4F,G, Figure S4C-E). IHC results also showed that the expression of ULK1 was significantly decreased in the tumors from the *NSD2*-knockdown group (Figure S4F-G). Moreover, we found that the expression of ULK1 was markedly increased in TNBC cells compared to that in normal MCF-10A cells (Figure S4H-J). Consistently, the expression of ULK1 was significantly increased in TNBC tumor tissues (Figure 4H,I) and positively correlated with NSD2 and H3K36me2 (Figure 4J).

To test whether NSD2 promotes ULK1 expression dependent on its H3K36 dimethylation activity, *NSD2*-knockdown cells were used to express wild-type *NSD2* or catalytic inactivating mutation (*NSD2*^Y1179A^). We found that re-expression of wild-type *NSD2* in *NSD2*-knockdown cells effectively rescued H3K36me2 and ULK1 expression, whereas ectopic expression of *NSD2*^*Y1179A*^ had no obvious effects on H3K36me2 and ULK1 expression (Figure 4K), suggesting that the H3K36 dimethylation activity of NSD2 is indispensable for its role in promoting ULK1 expression. We further determined whether NSD2 participates in the regulation of H3K36me2 modification of the *ULK1* gene. Chromatin immunoprecipitation (ChIP) showed that NSD2 and H3K36me2 were significantly enriched at ~ 0.8 kb to 1.2 kb (P4 and P5) upstream of the *ULK1* transcription start site (TSS) (Figure 4L,M). Moreover, our data showed that the levels of H3K36me2 modification at these loci in *NSD2*-knockdown MDA-MB-231 cells were significantly lower than those in control cells (Figure 4N). In contrast, overexpression of *NSD2* significantly increased the levels of H3K36me2 modification at the *ULK1* promoter (Figure 4O). We further generated luciferase reporters containing either *ULK1* wild-type (WT) or mutant promoter. Luciferase reporter analysis showed that overexpression of *NSD2* significantly increased WT promoter reporter activity, but it had little effect on the luciferase activity of mutant promoter reporter in MDA-MB-231 and BT549 cells (Figure 4P). Altogether, these results indicate that NSD2-mediated H3K36me2 results in a transcriptional activation of *ULK1*.

### NSD2 drives ULK1-induced autophagy in TNBC

**Figure 5.**
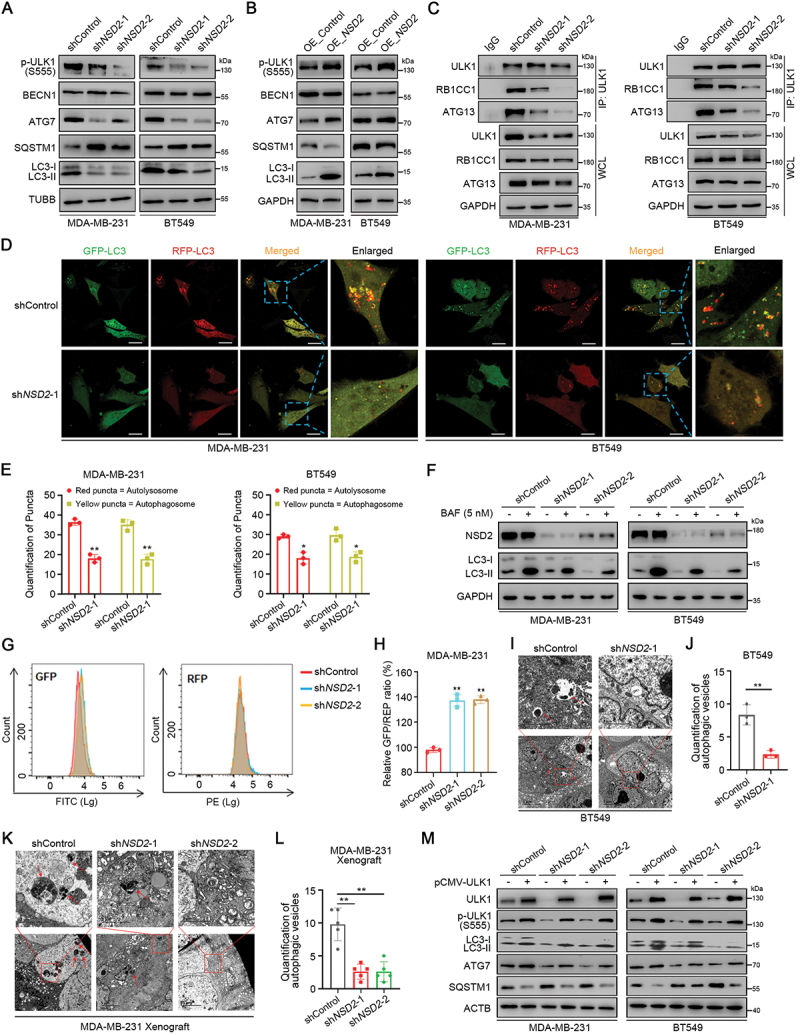
NSD2 drives ULK1-induced autophagy in TNBC. (A) MDA-MB-231 and BT549 cells were transfected with *NSD2* shRNA or control shRNA, the expression of p-ULK1 (S555), BECN1, ATG7, SQSTM1, and LC3B were measured by western blotting. (B) MDA-MB-231 and BT549 cells were transfected with *NSD2*-overexpressing vector or control vector, the expression of p-ULK1 (S555), BECN1, ATG7, SQSTM1, and LC3B were measured by western blotting. (C) Total protein extracts of MDA-MB-231 and BT549 cells with *NSD2* knockdown were subjected to IP using ULK1 antibody, followed by IB with ULK1, RB1CC1, and ATG13 antibodies. (D and E) Immunofluorescence (IF) staining with mRFP-GFP-LC3 in *NSD2*-knockdown MDA-MB-231 and BT549 cells. Red puncta signified autolysosomes and yellow puncta signified autophagosomes, scale bar: 25 μm (D). Quantification of LC3 puncta (E). (F) *NSD2*-knockdown MDA-MB-231 and BT549 cells or control cells were treated with 5 nM BAF for 24 h, the expression of NSD2 and LC3B were measured by western blotting. (G and H) *NSD2*-knockdown MDA-MB-231 cells and control cells were transfected with GFP-LC3-RFP-LC3ΔG probe, the autophagic flux were analyzed by flow cytometry, histograms of fluorescence intensity versus cell count (G) and the GFP:RFP fluorescence ratio (H) are shown. (I and J) Transmission electron microscopy (TEM) showing autolysosomes and autophagosomes in *NSD2*-knockdown BT549 cells (I). Quantification of autophagic vesicles (J). (K and L) TEM images showing autolysosomes and autophagosomes in *NSD2*-knockdown MDA-MB-231 ×enograft tumor (K). Quantification of autophagic vesicles (L). (M) *NSD2*-knockdown MDA-MB-231 and BT549 cells were transfected with pCMV-*ULK1* ORF vector or control vector, the protein levels of ULK1, p-ULK1 (S555), LC3B, ATG7, and SQSTM1 were measured by western blotting. Error bars represented the mean ± sem and the dots represented the value of each experiment. *p < 0.05, **p < 0.01.

Since ULK1 is an important autophagy regulator, we then explored the association between NSD2 expression and autophagy. In accordance with decreased ULK1 expression, the phosphorylation of ULK1 at Ser555 was significantly decreased in *NSD2*-knockdown MDA-MB-231 and BT549 cells (Figure 5A, Figure S5A). In contrast, overexpression of *NSD2* significantly increased ULK1 phosphorylation at Ser555 in MDA-MB-231 and BT549 cells (Figure 5B, Figure S5B). Importantly, we found that knockdown of *NSD2* inhibited autophagy initiation complex ULK1–ATG13–RB1CC1/FIP200 assembly (Figure 5C, Figure S5C). We further investigated the effects of NSD2 on the expression of autophagy-related factors involved in phagophore nucleation/expansion and the delivery of autophagic cargo to phagophores. Our results showed that knockdown of *NSD2* significantly decreased LC3B and ATG7 expression, and increased SQSTM1/p62 expression in MDA-MB-231 and BT549 cells (Figure 5A, Figure S5A). In contrast, overexpression of *NSD2* increased LC3B and ATG7 expression, while decreased SQSTM1 expression (Figure 5B, Figure S5B). Moreover, decreased LC3B expression was observed in *NSD2*-knockdown MDA-MB-231 tumor (Figure S5D-E).

We next detected autophagic flux in TNBC cells. Immunofluorescence assays showed that knockdown of *NSD2* resulted in a remarkable decrease in mRFP-GFP-LC3 dot accumulation (Figure 5D,E). Given that autophagy is a dynamic process with multiple steps and the change in LC3B protein level or LC3B puncta reflects both autophagosome biogenesis and degradation, we further confirmed the effect of NSD2 on autophagosome biogenesis upon inhibition of autophagosomal degradation by bafilomycin A_1_ (BAF). The results showed that treatment of BAF significantly increased LC3B accumulation in *NSD2*-knockdown MDA-MB-231 and BT549 cells (Figure 5F, Figure S5E), suggesting that the inhibition of autophagosome biogenesis in *NSD2*-knockdown TNBC cells is reversed by BAF. This phenomenon was further supported by the observation of the effect of autophagy inhibitor chloroquine (CQ) (Figure S5F-G). Next, we used the GFP-LC3B-RFP-LC3ΔG probe to evaluate autophagic flux. When overexpressed in the cells, this probe is specifically cleaved to form GFP-LC3 and RFP-LC3ΔG fragments. GFP-LC3 is conjugated with phosphatidylethanolamine (PE) and subsequently degraded by autophagy, whereas REP-LC3ΔG is stably present in the cytoplasm. Then, autophagic flux can be quantitatively estimated by determining the ratio of GFP and RFP fluorescence intensities [30]. We found that overexpression of *GFP-LC3B-RFP-LC3ΔG* in *NSD2*-knockdown MDA-MB-231 cells showed an increase of GFP signals and GFP:RFP ratio (Figure 5G,H), suggesting that knockdown of *NSD2* suppresses autophagic flux. Moreover, transition electron microscopy (TEM) revealed that knockdown of *NSD2* decreased the formation of autophagosomes in BT549 cells (Figure 5I,J). Similarly, we observed decreased autophagosomes in *NSD2*-knockdown MDA-MB-231 ×enograft when comparing to the control tumor (Figure 5K,L). Furthermore, we restored *ULK1* expression in *NSD2*-knockdown MDA-MB-231 and BT549 cells (Figure 5M, Figure S5I) to investigate the role of ULK1 in NSD2-induced autophagy. Our results showed that the effects of *NSD2* knockdown on p-ULK (S555), LC3B, ATG7, and SQSTM1 expression were almost completely reversed by *ULK1* overexpression (Figure 5M, Figure S5I), suggesting that NSD2 promotes autophagy via inducing ULK1 expression.

Previous studies suggested that activation of autophagy serves as a protective response under hypoxia in cancer cells [31]. We then analyzed the role of NSD2 on autophagy under hypoxic conditions. We found that hypoxia significantly increased the expression of ULK1, ATG7, and LC3B, whereas decreased the expression of SQSTM1 in MDA-MB-231 cells (Figure S5J-L). However, the effects of hypoxia on the expression of autophagy markers were partly reversed by specific knockdown of *NSD2* (Figure S5J-L), implying that NSD2 plays an important role in hypoxia-induced autophagy in TNBC cells. Taken together, all of the above results demonstrate that NSD2 drives ULK1-induced autophagy in TNBC cells.

### NSD2 promotes autophagy-associated TNBC progression

**Figure 6.**
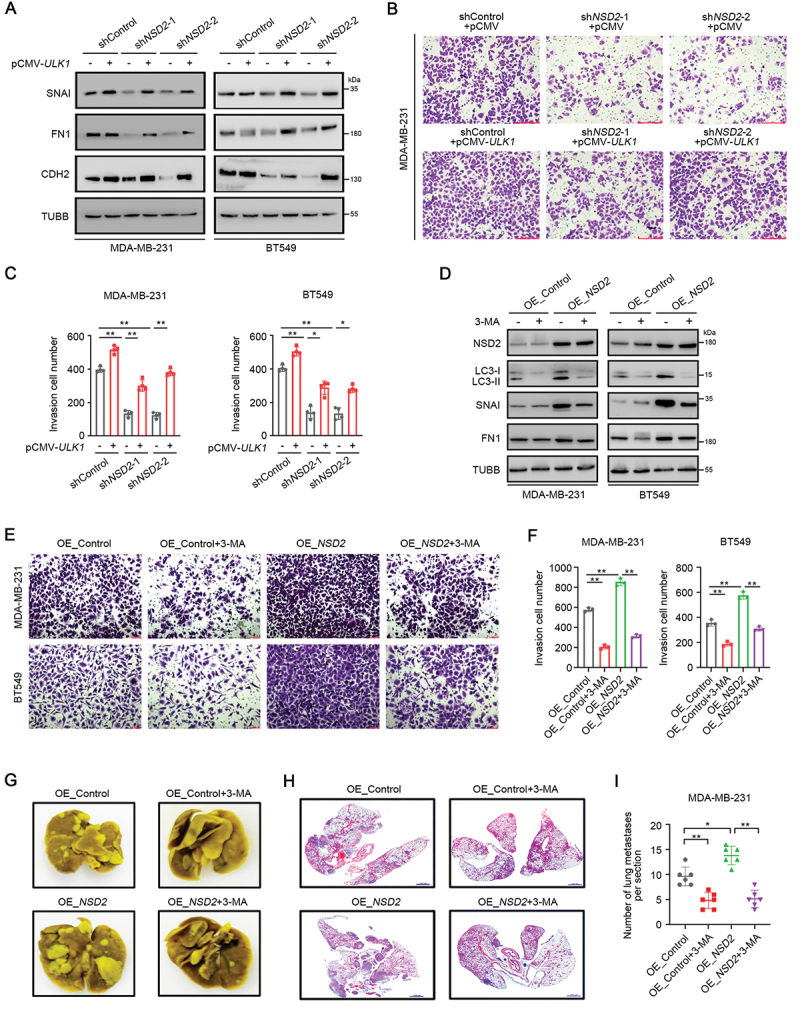
NSD2 promotes autophagy-associated TNBC progression. (A) *NSD2*-knockdown MDA-MB-231 and BT549 cells were transfected with pCMV-*ULK1* ORF vector or control vector, the protein levels of SNAI, FN1, and CDH2 were measured by western blotting. (B and C) *NSD2*-knockdown MDA-MB-231 cells were transfected with pCMV-*ULK1* ORF vector or control vector, cell invasion ability was measured by transwell assay. Scale bar: 200 μm. (D) MDA-MB-231 and BT549 cells transfected with *NSD2*-overexpressing vector or control vector were treated with autophagy inhibitor 3-MA (5 mM) for 24 h, the protein levels of NSD2, LC3B, SNAI, and FN1 were measured by western blotting. (E and F) MDA-MB-231 and BT549 cells transfected with *NSD2-*overexpressing vector or control vector were treated with autophagy inhibitor 3-MA (5 mM) for 24 h, cell invasion ability was measured by transwell assay. Scale bar: 100 μm. (G-I) A total of 1 × 10^6^ MDA-MB-231 cells stably transfected with *NSD2*-overexpressing vector or control vector were tail vein-injected into nude mice (*N* = 6 per group), followed by intraperitoneal (i.P.) injection of 3-MA (30 mg/kg/day) or vehicle control (water) for 42 days. Representative images showing lung nodules (G). Representative H&E images of lung tissues showing metastatic nodules, scale bar: 1000 μm (H). Average number of lung metastases nodules in the indicated groups (I). Error bars represented the mean ± sem and the dots represented the value of each experiment. *p < 0.05, **p < 0.01.

Previous reports have shown that autophagy is associated with malignancy in human tumors [32]. Given that NSD2 plays a more important role in promoting TNBC cell autophagy, we then investigated whether the tumor-promoting role of NSD2 is related with autophagy. Consistent with the reversal effect of *ULK1* overexpression on *NSD2* knockdown-mediated autophagy inhibition (Figure 5M), overexpression of *ULK1* caused the upregulation of CDH2, FN1, and SNAI in *NSD2*-knockdown MDA-MB-231 and BT549 cells (Figure 6A, Figure S6A). Importantly, transwell assays revealed that the reduced migration and invasion ability of *NSD2*-knockdown TNBC cells could be recovered by *ULK1* overexpression (Figure 6B,C, Figure S6B), suggesting that ULK1 contributes to NSD2-mediated TNBC progression. We further performed experiments with downregulating autophagy by the autophagy inhibitor 3-methyladenine (3-MA) in *NSD2*-overexpressing TNBC cells. We found that treatment with 3-MA significantly reduced LC3B accumulation (Figure 6D, Figure S6C). In particular, the expression of EMT markers were significantly increased in *NSD2*-overexpressing MDA-MB-231 and BT549 cells, while they were further reduced in the 3-MA-treated group (Figure 6D, Figure S6C). Consistently, the promoting effects of *NSD2* overexpression on cell migration and invasion were almost completely reversed by specific inhibition of autophagy in both MDA-MB-231 and BT549 cells (Figure 6E,F).

We further examined the role of ULK1-mediated autophagy in TNBC progression induced by *NSD2* overexpression *in vivo*. *NSD2*-overexpressing MDA-MB-231 cells were inoculated into the nude mice. After tumors formed, the tumor-bearing mice were treated with vehicle (Control) or 3-MA. As expected, treatment with 3-MA significantly reduced tumor growth (Figure S6D-E). Immunohistochemistry assays revealed that treatment of 3-MA resulted in a striking decreased expression of CDH2 and SNAI in the *NSD2*-overexpressing MDA-MB-231 tumor (Figure S6F-G). Furthermore, *NSD2*-overexpressing MDA-MB-231 cells were injected into tail veins of nude mice, followed by treatment of 3-MA. Notably, while *NSD2* overexpression markedly increased the metastasis of MDA-MB-231 cells, treatment with 3-MA attenuated this effect and caused a dramatic reduction of lung metastatic colonies in the MDA-MB-231/*NSD2* group (Figure 6G-I). Taken together, these data indicate that NSD2 promotes TNBC progression via ULK1-mediated autophagy activation.

### Targeting NSD2 inhibits TNBC autophagy, growth, and metastasis

**Figure 7.**
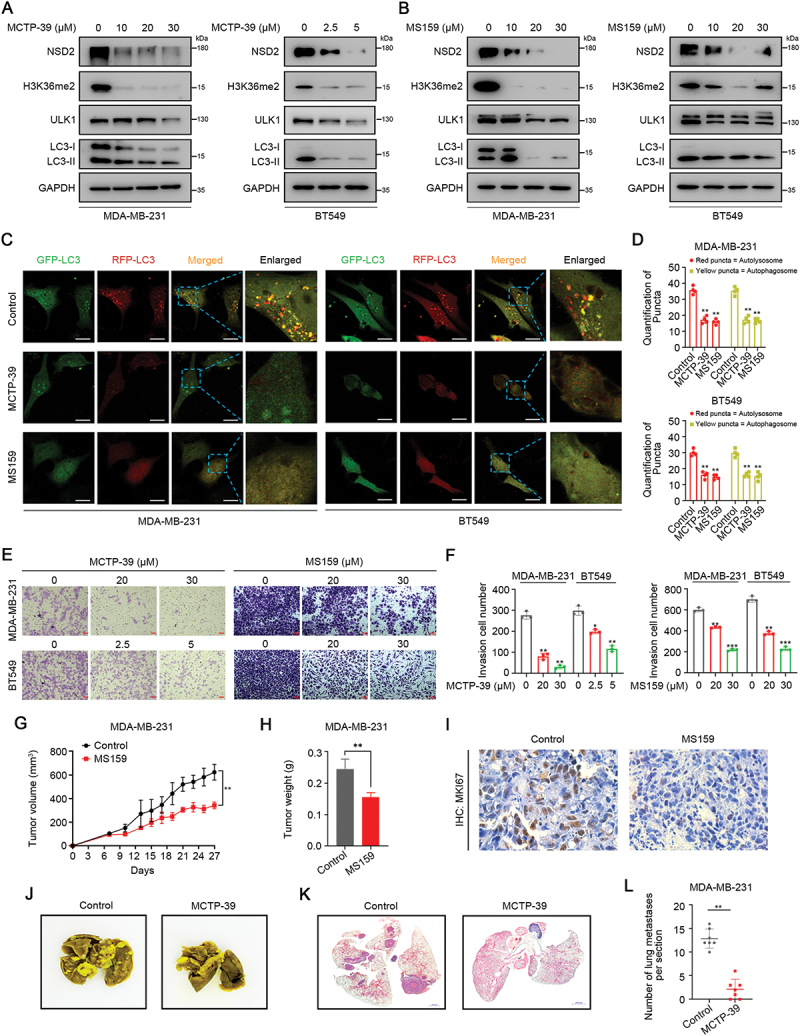
Targeting NSD2 inhibits TNBC autophagy, growth, and metastasis. (A) MDA-MB-231 and BT549 cells were treated with MCTP-39 at indicated concentrations for 48 h, the protein levels of NSD2, H3K36me2, ULK1, and LC3B were measured by western blotting. (B) MDA-MB-231 and BT549 cells were treated with MS159 at indicated concentrations for 48 h, the protein levels of NSD2, H3K36me2, ULK1, and LC3B were measured by western blotting. (C and D) MDA-MB-231 and BT549 cells were treated with MCTP-39 (30 μM) or MS159 (5 μM) for 48 h, immunofluorescence staining with mRFP-GFP-LC3 in cells. Red puncta signified autolysosomes and yellow puncta signified autophagosomes, scale bar: 25 μm (C). Quantification of LC3 puncta (D). (E and F) MDA-MB-231 and BT549 cells were treated with MCTP-39 or MS159 for 24 h, cell invasion ability was measured by transwell assay. Scale bar: 100 μm. (G-I) A total of 5 × 10^6^ MDA-MB-231 cells were inoculated subcutaneously into the female nude mice and palpable tumors were allowed to develop for 7 days, mice were randomly allocated into two groups (*N* = 5 per group): vehicle control (10% DMSO in 40% PEG300, 5% tween 80 and 45% saline), MS159 (25 mg/kg/day) via intraperitoneal (i.P.) injection for 21 days. Tumor size was measured at indicated time intervals and tumor volume was calculated (G). At the end of treatment, tumors were excised and tumor weights were measured (H). Tumor specimens were subjected to IHC staining for MKI67, scale bar: 50 μm (I). (J-L) A total of 1 × 10^6^ MDA-MB-231 cells were tail vein-injected into nude mice (*N* = 7 per group), followed by intraperitoneal (i.P.) injection of MCTP-39 (30 mg/kg/day) or vehicle control (10% DMSO in 20% SBE-β-CD) for 42 days. Representative images showing lung nodules (J). Representative H&E images of lung tissues showing metastatic nodules, scale bar: 1000 μm (K). Average number of lung metastases nodules of indicated groups (L). Error bars represented the mean ± sem and the dots represented the value of each experiment. *p < 0.05, **p < 0.01.

Given the tumor-promoting effects of NSD2 in TNBC, we took advantage of a pharmacological approach to suppress NSD2 expression and activity. MCTP-39 has been recently identified as potent inhibitors of NSD2 [23], and MS159 is a first-in-class NSD2 proteolysis targeting chimera/PROTAC degrader that effectively degraded NSD2 [33]. Accordingly, we found that treatment of MDA-MB-231 and BT549 cells with MCTP-39 potently downregulated NSD2 and H3K36me2 expression in a dose-dependent manner (Figure 7A, Figure S7A). Similar results were also obtained in MDA-MB-231 and BT549 cells treated with MS159 (Figure 7B, Figure S7B). Intriguingly, treatment with MCTP-39 or MS159 significantly reduced the expression of ULK1 and LC3B (Figure 7A,B, Figure S7A-B). Consistently, inhibition of NSD2 decreased the formation of autophagosomes in MDA-MB-231 cells (Figure S7C-D). Immunofluorescence assays showed that inhibition of NSD2 resulted in a remarkable decrease in mRFP-GFP-LC3 dot accumulation (Figure 7C,D), suggesting that the pharmacological inhibition of NSD2 decreases autophagy. Similar to *NSD2* knockdown, the specific inhibition of NSD2 significantly decreased TNBC cell migration and invasion (Figure 7E,F). To determine the antitumor activity of NSD2 inhibitor *in vivo*, we tested the effect of MS159 on tumor growth in the MDA-MB-231 cell-derived xenograft model. After tumors formed, the tumor-bearing mice were treated with vehicle (Control) or MS159. As expected, treatment with MS159 reduced tumor growth (Figure 7G,H). Immunohistochemistry assays revealed that treatment with MS159 resulted in a striking decrease of cell proliferation marker MKI67/Ki-67 in the tumor xenografts (Figure 7I). Next, we evaluated the effect of MCTP-39 on lung metastasis *in vivo* and observed a significant decrease in lung metastatic colonies in the mice treated with MCTP-39 as compared to those treated with control (Figure 7J-L). Moreover, all mice of each treatment group showed stable body weights, indicating that treatment with MS159 or MCTP-39 has no significant toxicity (Figure S7E-F). Taken together, these results demonstrate that pharmacological inhibition of NSD2 suppresses TNBC autophagy, growth, and metastasis.

## Discussion

Metastasis is a major barrier for effective cancer therapy [34]. TNBC is the most aggressive subtype of breast cancers with high probability of metastasis as well as lack of specific targets and targeted therapeutics. Therefore, understanding precise mechanisms underlying TNBC metastasis is imperative and effective treatments are emerging.

NSD2 is aberrant expression in a variety of cancers [16,22]. The oncogenic role of NSD2 was first discovered in multiple myeloma [14]. Emerging evidence also implied that NSD2 is implicated in cancer progression of several solid tumors [35,36]. For example, NSD2 plays an important role in promoting the metastatic behavior of prostate cancers [23,27,37], which is consistent with the fact that H3K36me2 modification is generally associated with EMT and cancer cell metastasis [38]. Several studies have elucidated the correlation between NSD2 and breast cancer progression and explored its regulatory mechanisms, implying a key role of NSD2 in promoting breast cancer progression [39]. For example, NSD2 acts as an essential regulator of estrogen receptor signaling and is a potential therapeutic target for endocrine-resistant breast cancer [40,41]. Wang *et al*. suggested that *NSD2* overexpression stimulates EGFR-AKT signaling and promotes TNBC cell resistance to the EGFR inhibitor gefitinib [42]. Our current data further our understanding of the function of NSD2 in promoting TNBC progression. We demonstrated that NSD2 is highly expressed and associated with aggressiveness and poor survival of TNBC. The role of NSD2 in TNBC metastasis was further confirmed via multiple TNBC models both *in vitro* and *in vivo*. Moreover, we demonstrated that inhibition of NSD2 by MCTP-39 or MS159 significantly suppresses autophagy, growth, and metastasis of TNBC, providing the possibility to develop novel therapeutic approaches for treatment of advanced TNBC by targeting NSD2.

Mechanistically, our data suggested that the NSD2-H3K36me2 axis promotes TNBC metastasis through regulation of ULK1-dependent autophagy. The function of NSD2 in cancer progression is largely dependent on its enzymatic activity [24]. NSD2 catalyzes the dimethylation of H3K36, a permissive mark associated with active gene transcription [43]. We discovered that NSD2 engages the promoter of *ULK1* and displayed elevated enrichment at the regions (−0.8 kb ~ −1.2 kb), which in turn increases the *ULK1* promoter activity. Moreover, re-expression wild-type *NSD2* but not *NSD2*^Y1179A^ catalytic inactivating mutation effectively rescues H3K36me2 and ULK1 expression in *NSD2*-knockdown cells. These studies support the notion that NSD2-mediated H3K36me2 maintains ULK1 overexpression in TNBC cells through its impacts on *ULK1* gene transcription.

Previous studies have suggested the crucial function of ULK1 in autophagosome formation through phosphorylating multiple downstream factors [44]. We further explored the link between NSD2 and autophagy in TNBC. We observed that knockdown of *NSD2* decreases the phosphorylation level of ULK1, thereby inhibiting autophagy initiation complex ULK1-ATG13-RB1CC1 assembly. LC3 is involved in autophagosome biogenesis and is widely considered as a classical marker of autophagy [45,46]. Our result showed that *NSD2* knockdown leads to a markedly decreased LC3B expression, which results in a suppression of autophagosome formation in TNBC cells. We also observed that *NSD2* knockdown results in a remarkable decrease in autophagic flux. Thus, we are the first to demonstrate that NSD2-mediated H3K36me2 acts as a novel epigenetic regulator of autophagy during TNBC progression. Furthermore, DCN (decorin), a member of the class I small leucine-rich proteoglycans, is significantly increased in *NSD2*-overexpressing TNBC cells based on RNA-seq data. Although DCN has been shown to induce autophagy in endothelial cells, ectopic expression of DCN is shown to reduce extracellular matrix degradation and inhibit cell invasion [47,48,49]. Therefore, the role of DCN in NSD2-induced autophagy and metastasis should be further explored.

Accumulating evidence highlighted the role of autophagy in cancer development and progression [50]. However, autophagy plays a double-faced role in these processes. While autophagy may be a survival pathway to prevent tumor initiation and suppress cancer progression during early tumorigenesis, it is essential for cancer progression by producing nutrient substances and releasing ATP during the advanced stages of cancer [51]. Autophagy also plays a complex and stage-specific role and promotes multiple steps during cancer metastasis [52,53]. Autophagy may act as a tumor metastasis suppressor by preventing tumor necrosis and restricting inflammatory cell infiltration during the early stage of metastasis [54]. However, autophagy has been shown to be involved in modulating tumor cell motility and invasion, cancer stem cell differentiation, epithelial-to-mesenchymal transition, and escape from immune surveillance, with emerging functions in establishing the pre-metastatic niche and other aspects of metastasis [52]. Using the autophagy marker, LC3B, previous studies have identified an association between increased autophagy and metastasis in several types of cancer including breast cancer metastasis [8,55]. Autophagy promotes focal adhesion disassembly and cell motility of metastatic tumor cells through the direct interaction of PXN (paxillin) with LC3 [56]. In agreement with these reports, we demonstrated the essential role of autophagy activation by NSD2-mediated H3K36me2 in promoting TNBC metastasis. First, *ULK1* overexpression reverses the expression of EMT marker and increases cell migration and invasion ability in *NSD2*-knockdown TNBC cells. Second, treatment with the autophagy inhibitor 3-MA, blocking the formation of autophagosomes, reverses *NSD2* overexpressing-induced EMT phenotypes, thereby attenuating cell migration and invasion *in vitro* and reducing NSD2-induced lung metastasis *in vivo*. All of this evidence supported the notion that NSD2-mediated H3K36me2 promotes autophagy-associated metastasis in TNBC. However, one question pertaining to the regulation mechanism on TNBC metastasis by the “NSD2-H3K36me2-ULK1-autophagy” axis still remains unknown. We previously demonstrated that the FOXO1-ULK1 axis promotes histone deacetylase inhibitor-induced expression of SNAI/snail by increasing SMAD2 and SMAD3 phosphorylation and nuclear translocation [57]. We speculate that this mechanism applies to the “NSD2-H3K36me2-ULK1-autophagy” axis as well, in which the ULK1-triggered autophagy can activate the SMAD2- or SMAD3-SNAI signaling pathway, thereby promoting TNBC metastasis. Nevertheless, we hope this question will be addressed in the near future.

Previous studies of NSD2 have focused on its function and the mechanism of downstream regulation. However, the possible upstream regulation of NSD2 remains unclear. In the present study, we observed that hypoxia induces NSD2 expression in a HIF1A-dependent manner, and is positively correlated between HIF1A and NSD2 or H3K36me2, implying that *NSD2* is a novel target gene of HIF1A. Hypoxia is an intrinsic property of the tumor microenvironment [58]. The cellular response to hypoxia, followed by activation of HIF, has been reported to be emerging as an important mechanism promoting TNBC aggressiveness, metastasis, and poor prognosis [59]. Meanwhile, cancer cells respond to hypoxia by adapting their metabolism and function through a number of hypoxia-associated pathways including autophagy to promote tumor growth, aggressiveness and metastasis [60,61]. In accordance with hypoxia-induced NSD2 expression, knockdown of *NSD2* decreases hypoxia-induced autophagy and cell invasion of TNBC cells, suggesting that NSD2 contributes to hypoxia-induced autophagy and autophagy-related metastasis of TNBC.

In summary, we reveal that NSD2 is a functional driver of TNBC metastasis. NSD2-mediated H3K36me2 transcriptionally activates the expression of ULK1, thereby promoting autophagy-associated metastasis of TNBC. Our data strongly support that the NSD2-ULK1-autophagy axis may be a novel target for developing more effective strategies for the treatment of metastatic TNBC.

## Materials and methods

### Cell culture and reagents

Human breast cancer cell lines MDA-MB-231(HTB-26), BT549 (HTB-122), BT-20 (HTB-19), ZR-75-30 (CRL-1504), SKBR3 (HTB-30), MDA-MB-453 (HTB-131), T47D (HTB-133), MCF-7 (HTB-22), ZR-75-1 (CRL-1500), and human breast epithelial cell line MCF-10A (CRL-10317) were obtained from American Type Culture Collection (ATCC). The breast cancer cells were cultivated in Dulbecco’s modified Eagle’s medium (DMEM; Gibco, C11995500BT) or RPMI 1640 medium (Gibco, C11875500BT) supplemented with 10% FBS (Gibco 10,270,106) and 1% penicillin-streptomycin (Gibco 15,140,122). MCF-10A cells were cultured in DMEM/F-12 medium (Gibco, C11330500BT) supplemented with 5% horse serum (Sigma, 12449C), 100 ng/mL cholera toxin (Sigma, C8052), 500 ng/mL hydrocortisone (Sigma, 3867), 20 ng/mL EGF (epidermal growth factor; Peprotech, AF-100-15), and 0.01 mg/mL insulin (Sigma, I3536). All cell lines were maintained at 37°C in a humidified atmosphere containing 5% CO_2_. Cell lines were authenticated using short tandem repeat (STR) profiling every 6 months. 3-MA (S2767)-PEG300 (S6704), Tween 80 (S6702), SBE-β-CD (S4592) were purchased from Selleck. LW6 (HY-13671), chloroquine (CQ; HY-17589), bafilomycin A_1_ (BAF; HY-100558), MCTP-39 (HY-120536), and MS159 (HY-151101) were purchased from MCE.

### Patients and samples

Primary tumor specimens were obtained from 114 patients diagnosed with breast cancer who underwent complete resection in the Affiliated Tumor Hospital of Guangzhou Medical University between 2010 and 2015. Follow-up information was obtained from reviews of the patient’s medical records. This study was approved by the Ethics Committee of the Affiliated Tumor Hospital of Guangzhou Medical University (2021-G07) and performed in accordance with the Declaration of Helsinki. Written informed consent was provided by all patients.

### Quantitative RT-PCR

Total RNA was isolated using a TRIzol reagent (Invitrogen 15,596,018). First-strand cDNA was synthesized using a RevertAid™ First Strand cDNA Synthesis Kit (Thermo Fisher Scientific, K1622). Quantitative real-time PCR was performed using a LightCycler^@^ 96 Instrument (Roche). Relative RNA abundances were calculated by the standard 2^−ΔΔCt^ method. The primers are listed in Table S1.

### Western blotting

Cells were lysed in RIPA Buffer (Thermo Fisher Scientific 89,901) containing 100× Protease & Phosphatase Inhibitor Cocktail (Thermo Fisher Scientific 78,440) and centrifuged at 12,000 × g for 30 min at 4°C. Protein lysates (30–50 µg) were separated on 10% or 12% SDS-polyacrylamide gels and transferred to 0.22 μm PVDF membranes (Millipore, ISEQ00010). Membranes were blocked with 5% skim milk powder in PBST, and probed with primary antibodies at 4°C overnight, including anti-NSD2 (1:1000), anti-H3K36me2 (1:1000), anti-CDH2 (1:1000), anti-FN1 (1:1000), anti-SNAI (1:500), anti-ULK1 (1:1000), anti-p-ULK1 (1:400), anti-BECN1 (1:5000), anti-SQSTM1 (1:5000), anti-ATG7 (1:1000), anti-LC3B (1:1000), anti-RB1CC1 (1:1000), anti-ATG13 (1:1000), anti-HIF1A (1:1000), anti-GAPDH (1:5000), anti-ACTB/β-actin (1:5000) and anti-TUBB/β-tubulin (1:1000) antibodies. Next, membranes were incubated with appropriate horseradish peroxidase-coupled secondary antibodies (Thermo Fisher Scientific; 31430, 31460) for 1 h. Visualization was performed using enhanced chemiluminescence (ECL) reagents (Thermo Fisher Scientific 34,580). Blot images were quantified by ImageJ software. Primary antibodies used in this study are listed in Table 1.

**Table 1.** Antibodies used in this study.

Antibodies	Source	Catalog#	
NSD2/WHSC1 antibody	Abcam	ab75359	For WB, ChIP
NSD2/WHSC1 antibody	Sigma	HPA015801	For IHC
H3K36me2 antibody	Abcam	ab9049	For WB, IHC, IF, ChIP
ACTB/β-actin antibody	Sigma	A5441	For WB
GAPDH antibody	Proteintech	60004–1-Ig	For WB
FN1 (fibronectin 1) antibody	Cell Signaling Technology	26836S	For WB, IHC
CDH2/N-cadherin antibody	Cell Signaling Technology	13116S	For WB, IHC
SNAI/snail antibody	Cell Signaling Technology	3879S	For WB, IHC
ULK1 antibody	HUABIO	ET1704–63	For WB
ULK1 antibody	Invitrogen	PA5–109212	For IHC, IF, IP
p-ULK1 (S555) antibody	Cell Signaling Technology	5869S	For WB
BECN1/Beclin-1 antibody	Proteintech	11306–1-AP	For WB
ATG7 antibody	HUABIO	ET1610–53	For WB
SQSTM1/p62 antibody	Proteintech	18420–1-AP	For WB
LC3B antibody	Abcam	ab192890	For WB, IHC
RB1CC1/FIP200 antibody	HUABIO	HA601106	For WB
ATG13 antibody	Cell Signaling Technology	13468S	For WB
TUBB/β-tubulin antibody	Cell Signaling Technology	86298S	For WB
MKI67/Ki-67 antibody	Cell Signaling Technology	9027S	For WB
HIF1A/HIF-1α antibody	Cell Signaling Technology	48085S	For WB
HIF1A/HIF-1α antibody	Sigma	MAB5382	For IHC
Goat anti-Mouse IgG (H+L) Secondary Antibody, HRP	Thermo Fisher Scientific	31430	For WB
Goat anti-Rabbit IgG (H+L) Secondary Antibody, HRP	Thermo Fisher Scientific	31460	For WB
Alexa Fluor 488 Goat anti-Rabbit IgG	Invitrogen	A11008	For IF
Alexa Fluor 594 Goat anti-Rabbit IgG	Invitrogen	A11012	For IF
IgG from rabbit serum	Sigma	I5006	For ChIP
IgG from mouse serum	Sigma	I5381	For ChIP

### Immunohistochemistry

Paraffin-embedded sections of clinical breast cancer tissues, tumor tissues from mouse xenograft models, and mouse lung tissues were subjected to immunohistochemistry. Briefly, the sections were deparaffinized in xylene, rehydrated with graded alcohol, and microwaved in 10 mm sodium citrate (pH 6.0) for 20 min. Hydrogen peroxide (0.3%) was applied to block endogenous peroxide activity. After incubation with 10% normal goat serum (Sigma, G9023), the sections were incubated with primary antibodies at 4°C overnight, including anti-NSD2 (1:50), anti-H3K36me2 (1:100), anti-CDH2 (1:50), anti-FN1 (1:100), anti-SNAI (1:25), anti-ULK1 (1:50), anti-LC3B (1:100), anti-MKI67 (1:100), and anti-HIF1A (1:50) antibodies. Then, the sections were washed 3 times with PBS (Beyotime, ST476) after incubation. Secondary antibodies were stained at room temperature for 2 h. Next, the sections were visualized by a DAB visualization kit (Maixin Bio, DAB-1031), and then counterstained with hematoxylin. IHC staining was evaluated by two independent pathologists. IHC score was calculated based on both the extent and the intensity of staining. The staining extent was scored as 0, 0–5%; 1, 5–25%; 2, 26–50%; 3, 51–75%; and 4, 76–100% according to the percentage of positively stained cells. The intensity of staining was scored as 0 (negative), 1 (weak), 2 (moderate), and 3 (strong). Specimens with the final scores ≥ 6 were defined as high expression and specimens with the final scores < 5 were defined as low expression. Primary antibodies used in this study are listed in Table 1.

### Plasmid and lentiviral transfection

Lentiviral particles for *HIF1A* shRNA (Y14908), *NSD2* shRNA (Y14909, Y14910) and control shRNA (GL427NC) were obtained from Shanghai OBio Technology. Cells were infected with lentiviruses for 48 h and then treated for 2 weeks with 2 μg/mL puromycin (Selleck, S7417). pCMV-*ULK1* ORF vector (EX-L5257), pCMV-*NSD2* ORF vector (EX-W0268), pCMV-*NSD2*^*Y1179A*^-mutant vector (CS-W0268), and control vector (EX-NEG) were obtained from GeneCopoeia Company. For cell transfection, TurboFect Transfection Reagent (Thermo Fisher Scientific, R0533) was used according to the manufacturer’s instructions. After 48 h of infection, stably expressing cells were selected in complete medium containing 2 μg/mL puromycin or 2 mg/mL G418 (Selleck, S3028).

### Transwell invasion assay

For invasion assays, cells were resuspended at 4 × 10^4^ cells/well in serum-free medium. A total of 200 μL of the cell suspension was seeded in the Matrigel-coated chamber (Corning 354,480), while a total of 750 μL of complete medium was added into the lower chamber. After 48 h with or without drug treatment, noninvasive cells and Matrigel were removed with a cotton swab. Invaded cells were fixed with methanol and then stained with crystal violet. Invaded cells were imaged and counted.

### Wound healing assay

Cells were cultured to full confluence in 6-well plates. Then, the monolayer of cells was scraped with a sterile pipette. The wounded region was observed with a microscope, and images were taken at 0, 24, and 48 h.

### Animal experiments

All animal works were performed in accordance with protocols approved by the Animal Experimentation Ethics Committee of Guangzhou Medical University (S2022–397).

For breast cancer xenograft models, a total of 5 × 10^6^ cells were injected subcutaneously into 4-week-old female BALB/c nude mice. Tumor growth was monitored by calipers, and tumor volume was calculated using the formula: V = 1/2 × larger diameter × (smaller diameter)^2^. At the end of the studies, the mice were sacrificed, and tumors were dissected for further IHC and immunofluorescence analysis.

For mouse models of lung metastasis, a total of 1 × 10^6^ cells were injected into the tail vein of 4-week-old female BALB/c nude mice. After 6 weeks, mice were sacrificed. The lungs were stained with Bouin’s fixative solution (Sigma, HT10132), paraffin-embedded and sliced. Lung sections were stained with hematoxylin and eosin (H&E). The number of metastatic colonies in the lungs was counted.

For orthotopic mouse models, a total of 1 × 10^5^ 4T1 cells were injected into the mammary fat pads of 4-week-old female BALB/c nude mice. Tumor growth was monitored by calipers, and the volume was calculated. After 16 days, mice were sacrificed. The lungs were fixed in 4% paraformaldehyde, paraffin-embedded and sliced. Lung sections were stained with H&E. The number of metastatic colonies in the lungs was counted.

### Co-immunoprecipitation

Cells grown in 10-cm plates were washed twice in ice-cold PBS and then lysed in IP Lysis Buffer (Thermo Fisher Scientific 87,787) supplemented with 100× Protease & Phosphatase Inhibitor Cocktail. Lysates were centrifuged at maximum speed at 4°C for 20 min. Clarified lysates were then incubated with 1 μg ULK1 antibody with rotation at 4°C overnight. The protein A/G magnetic beads (Thermo Fisher Scientific 88,802) were mixed with lysates for 1 h with rotation. Finally, the immunoprecipitates were washed three times in IP Lysis Buffer before eluted with SDS-PAGE sample buffer and subjected to western blotting analysis with indicated antibodies.

### Immunofluorescence staining

For tumor tissues from mouse xenograft models, samples were fixed in 4% paraformaldehyde at 4°C overnight. After washes, sink the samples in 10% sucrose solution for 4 h, 15% sucrose solution for 4 h, and 20% sucrose solution at 4°C overnight. The next day, samples were embedded in OCT (SAKURA, 4583). Samples were sliced into 4-μm sections and incubated with primary antibody against H3K36me2 at 1:500. For adherent cells, cells were fixed with 4% paraformaldehyde for 30 min. Then, fixed cells were washed and permeabilized with 0.1% Triton X-100 (Sigma, X100) for 10 min. After blocking in 5% BSA (Sigma, V900933) buffer for 1 h, the cells were incubated with primary antibody against ULK1 (Invitrogen) at 1:200 at 4°C overnight. The cells/sections were subsequently incubated with fluorescent secondary antibodies (Alexa Fluor 488 goat anti-rabbit IgG, Alexa Fluor 594 goat anti-rabbit IgG, at 1:1000 each) for 1 h. The nuclei were stained with DAPI (Thermo Fisher Scientific 62,248). Images were acquired using a Zeiss LSM710 confocal microscope at 200× magnification. Images were quantified by ImageJ software.

### RNA sequencing

Total RNA was extracted from *NSD2*-overexpressing MDA-MB-231 cells and control MDA-MB-231 cells from three independent biological replicates. RNA high throughput sequencing was performed by Cloud-Seq Biotech (Shanghai, China). Paired-end reads were harvested from an Illumina NovaSeq 6000 instrument, and were quality controlled by Q30. Fragments per kilobase of exon per million fragments mapped (FPKMs) was calculated as the expression profiles of mRNA using Cuffdiff software, and fold change and *p*-value were calculated based on FPKM. Differentially expressed mRNAs were identified. GO and Pathway enrichment analysis were performed based on the differentially expressed mRNAs.

### Chromatin immunoprecipitation

The ChIP assay was performed using a SimpleChIP® Enzymatic Chromatin IP Kit (Cell Signaling Technology, 9003S). Approximately 5 × 10^6^ cells were crosslinked with formaldehyde at final concentration of 1% for 5 min at room temperature and then quenched with 125 mm glycine. The nuclei preparation was conducted according to the manufacturer’s instructions. The digested chromatin was incubated with 3 μg anti-H3K36me2, anti-NSD2 (Abcam) or anti-IgG (Sigma, I5006 and I5381) antibodies at 4°C overnight. ChIP-Grade Protein G Magnetic Beads were then added into the lysates the following morning and incubated for 2 h. Purified DNA fragments were used for qPCR amplification with specific primers. The primers used are listed in Table S1.

### Double luciferase reporter assay

Double luciferase reporter gene assay was performed to investigate *ULK1* promoter activities mediated by NSD2. The wild-type and mutant *ULK1* promoter with the 5’-deleted region −1156/-1014 and −972/-870 relative to the transcription start site (TSS) were cloned into the *GLuc* Promoter Reporter plasmid (GeneCopoeia, CS-HPRM44998-PG04), separately. Next, the wild-type and mutant *ULK1* promoter plasmids were respectively transfected into *NSD2*-overexpressing cells and control cells using TurboFect Transfection Reagent for 48 h. GLuc and SEAP luciferase activities were measured using the Secrete-Pair™ Dual Luminescence Assay Kit (Genecopoeia, LF031). Luminescence was read on a BioTek Synergy 2 microplate reader. Relative luciferase activity was normalized to SEAP activity.

### Autophagic flux assay

To evaluate tandem fluorescent LC3 puncta, cells were transfected with *mRFP-GFP-LC3* plasmid (Addgene 21,074; deposited by Tamotsu Yoshimori) for 48 h. The fluorescence signals of transfected cells were sequentially acquired in single optical sections with a Zeiss LSM710 confocal microscope at 400× magnification. The relative fluorescence intensity values were determined with ImageJ software.

To measure autophagic flux, cells were transfected with *pMRX-IP-GFP-LC3-RFP-LC3ΔG* plasmid (Addgene 84,572; deposited by Noboru Mizushima) for 48 h. The GFP and RFP fluorescence signals were detected by BD FACSCanto II Flow Cytometry System equipped with a 488-nm laser and 561-nm laser. Data were processed with FlowJo v10.8.2 software.

### Transmission electron microscopy

Cells or xenograft tumor tissues were prefixed with 2.5% glutaraldehyde in 0.1 M phosphate buffer at 4°C overnight, then post-fixed in 1% osmium tetroxide, dehydrated in a graded series of ethanol and acetone and embedded in resin. Ultrathin sections were doubly stained with uranyl acetate and viewed with a Hitachi HT-7800 transmission electron microscope.

### Statistical analysis

Statistical analyses were conducted using GraphPad Prism 9.0 software. Comparisons between groups were analyzed by the *t*-test and χ2 test. Results were shown as the mean ± S.D. of multiple independent experiments. All experiments were performed at least three times. *p* < 0.05 was considered statistically significant.

## Supplementary Material

Supplementary Material revised 20250309 R6.docx

## Data Availability

The datasets used and analyzed during the current study are available from the corresponding author on reasonable request.
