# Supplementary material for: H3K36me2 methyltransferase NSD2/WHSC1 promotes triple-negative breast cancer metastasis via activation of ULK1-dependent autophagy
Source: Autophagy. 2025 Mar 25;21(8):1824–42. doi: 10.1080/15548627.2025.2479995 (PMC12283020; doi:10.1080/15548627.2025.2479995)
Supplement: Supplementary Material revised 20250309 R6.docx [file KAUP_A_2479995_SM4965.docx]

**Supplementary Material**

**H3K36me2 methyltransferase NSD2/WHSC1 promotes triple-negative breast cancer metastasis through activation of ULK1-dependent autophagy**

Danyang Chen^a#^, Xiaohui Chen^b#^, Mingqiang Yang^a#^, Qiunuo Li^b^, Shaojuan Weng^a^, Siyue Kou^a^, Xi Liu^c^*, Guanmin Jiang^b^*, Hao Liu^a^*


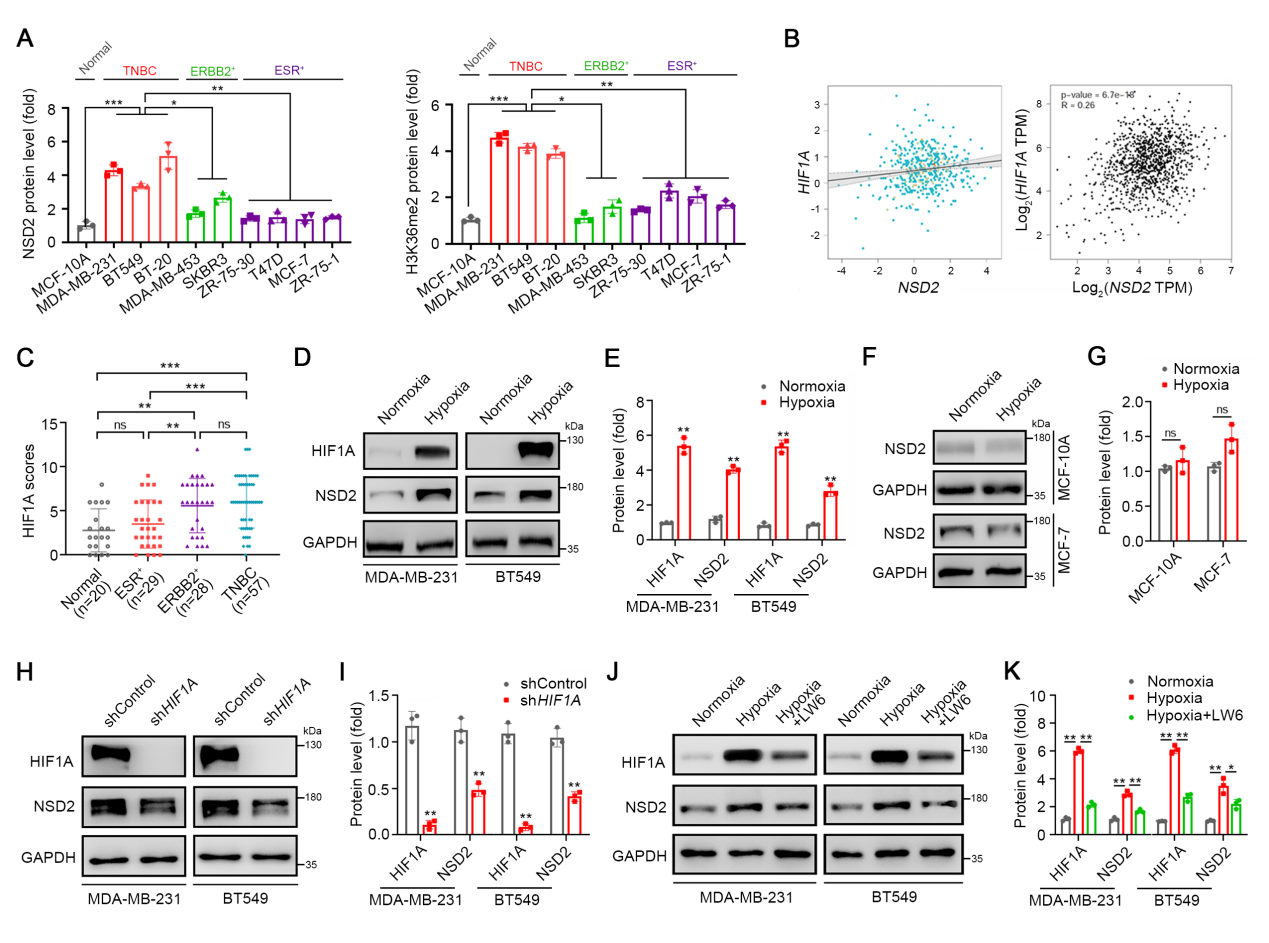


**Figure S1.** NSD2 is upregulated in TNBC and is associated with poor survival. (**A**) Quantification of western blotting images in (Figure 1B). (**B**) Correlation between mRNA expression of *HIF1A* and *NSD2* in the bc-GenExMiner v5.0 (Left) and GEPIA database (Right). (**C**) IHC staining scores for HIF1A in adjacent normal tissues and breast cancer tissues. (**D**) MDA-MB-231 and BT549 cells were cultured under normoxia (20% O_2_) or hypoxia (1% O_2_), the expression of HIF1A and NSD2 were measured by western blotting. (**E**) Quantification of western blotting images in (D). (**F**) MCF-10A and MCF-7 cells were cultured under normoxia (20% O_2_) or hypoxia (1% O_2_), the expression of NSD2 was measured by western blotting. (**G**) Quantification of western blotting images in (F). (**H**) MDA-MB-231 and BT549 cells were transfected with *HIF1A* shRNA or Control shRNA, the expression of NSD2 was measured by western blotting. (**I**) Quantification of western blotting images in (H). (**J**) MDA-MB-231 cells following LW6 treatment (10 μM) or vehicle were cultured under normoxia (20% O_2_) or hypoxia (1% O_2_), the expression of NSD2 was measured by western blotting. (**K**) Quantification of western blotting images in (J). Error bars represented the mean ± SEM and the dots represented the value of each experiment. * *p* < 0.05, ** *p* < 0.01.


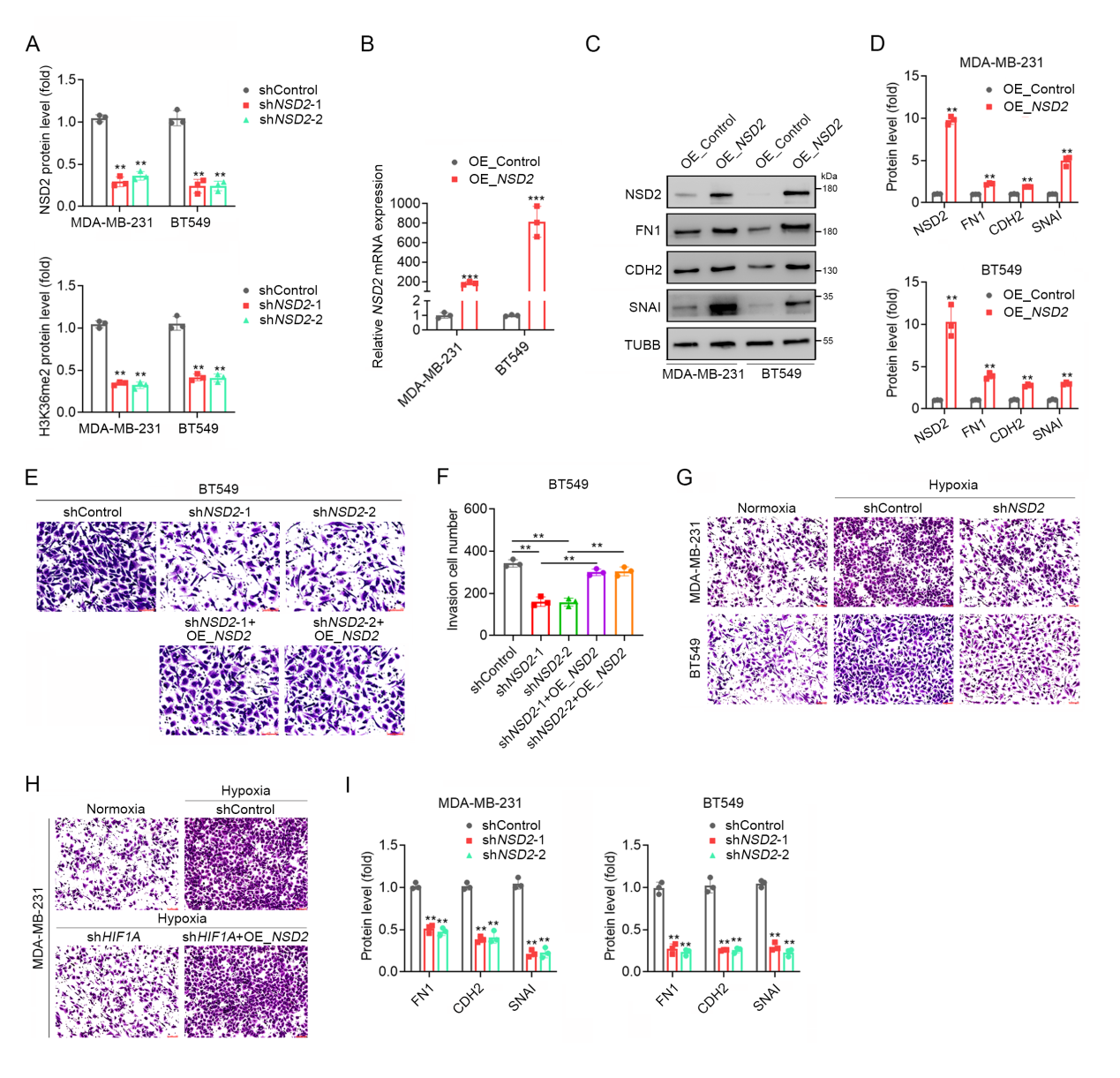


**Figure S2**. NSD2 promotes TNBC migration and invasion *in vitro*. (**A**) Quantification of western blotting images in (Figure 2B). (**B-D**) MDA-MB-231 and BT549 cells were stably transfected with *NSD2*-overexpressing vector or control vector, mRNA expression of *NSD2* was measured by qRT-PCR (**B**). Protein expression of NSD2, FN1, CDH2, and SNAI were measured by western blotting (**C**). Quantification of western blotting images in (C) (**D**). (**E and F**) *NSD2*-knockdown BT549 cells were stably transfected with *NSD2*-overexpressing vector, cell invasion ability was measured by transwell assay. Scale bar: 100 μm. (**G**) *NSD2*-knockdown MDA-MB-231 cells and control cells were cultured under normoxia (20% O_2_) or hypoxia (1% O_2_), cell invasion ability was measured by transwell assay. Scale bar: 100 μm. (**H**) *HIF1A*-knockdown MDA-MB-231 cells were cultured under normoxia (20% O_2_) or hypoxia (1% O_2_) after transfecting with *NSD2*-overexpressing vector or control vector, cell invasion ability was measured by transwell assay. Scale bar: 100 μm. (**I**) Quantification of western blotting images in (Figure 2N). Error bars represented the mean ± SEM and the dots represented the value of each experiment. * *p* < 0.05, ** *p* < 0.01, *** *p* < 0.001.


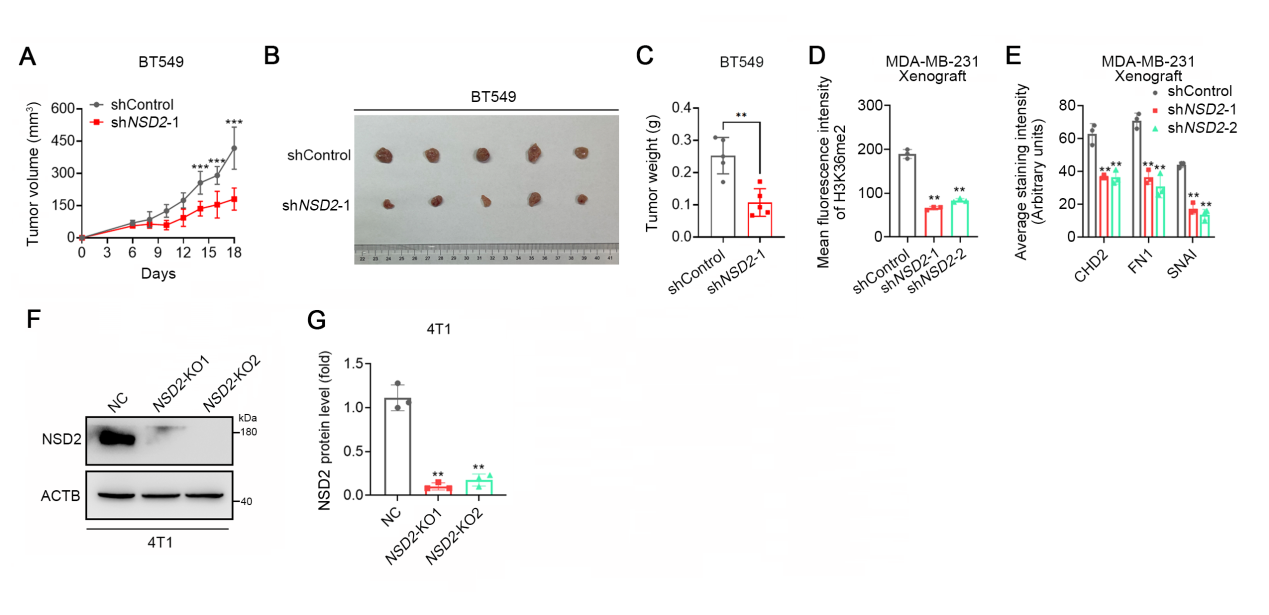


**Figure S3.** NSD2 promotes TNBC metastasis *in vivo*. (**A-C**) 5 × 10^6^ BT549/*NSD2* shRNA or BT549/Control shRNA cells were inoculated subcutaneously into the female nude mice (N = 5 per group). Tumor size was measured at indicated time intervals and tumor volume was calculated. Tumor growth curves were plotted using average tumor volume within each experimental group at the set time points (**A**). At the end of treatment, tumors were excised and imaged (**B**). Tumor weights were measured (**C**). (**D**) Quantification of immunofluorescence images in (Figure 3D). (**E**) Quantification of western blotting images in (Figure 3E). (**F and G**) 4T1 cells were stably transfected with *NSD2* sgRNA or control sgRNA, the expression of NSD2 was measured by western blotting (**F**). Quantification of western blotting images (**G**). Error bars represented the mean ± SEM and the dots represented the value of each experiment. ** *p* < 0.01, *** *p* < 0.001.


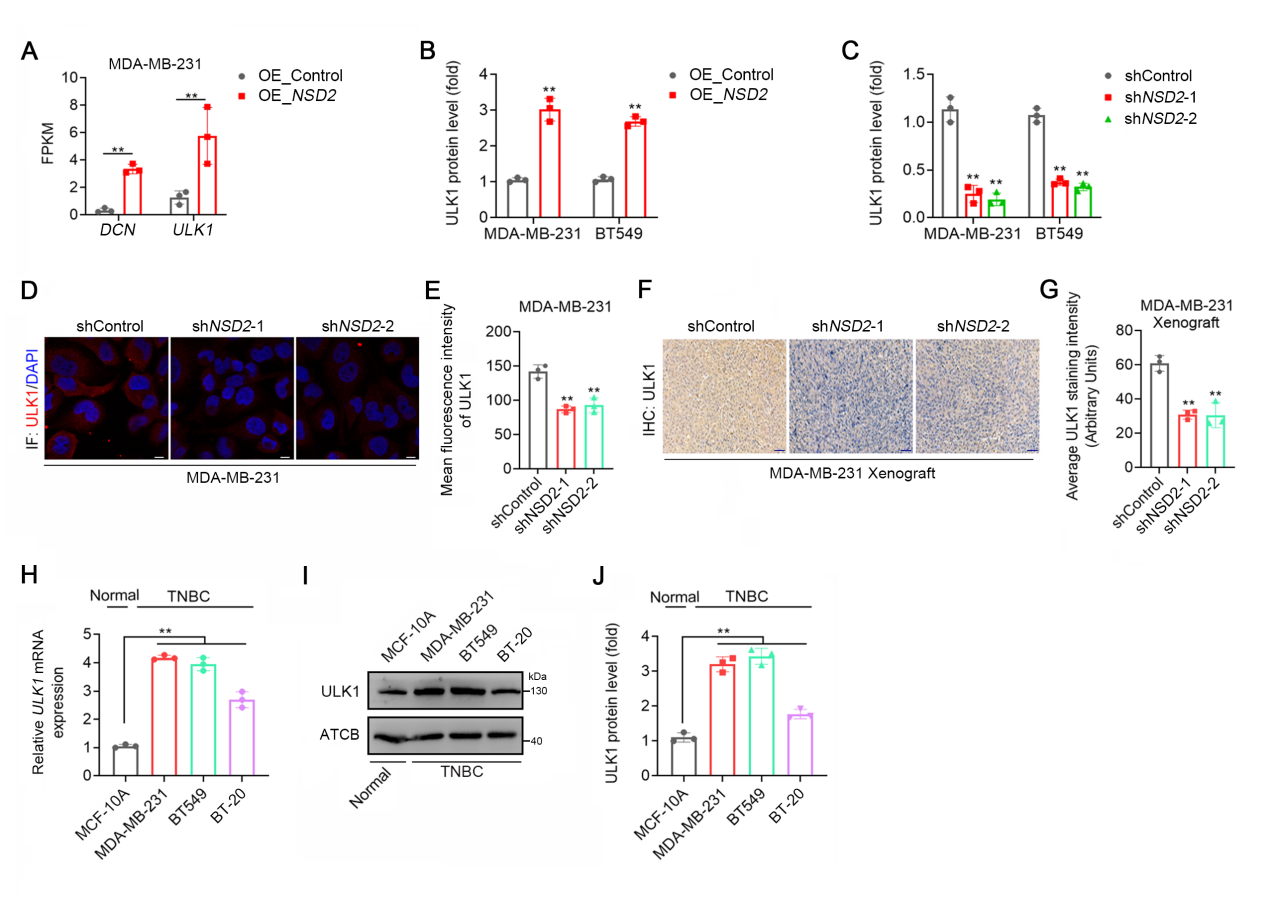


**Figure S4.** NSD2-mediated H3K36me2 promotes ULK1 expression in TNBC. (**A**) Average expression of *DCN* and *ULK1* in *NSD2*-overexpressing MDA-MB-231 cells normalized to control cells were determined by RNA-seq. (**B**) Quantification of western blotting images in (Figure 4E). (**C**) Quantification of western blotting images in (Figure 4G). (**D and E**) MDA-MB-231 cells were stably transfected with *NSD2* shRNA or control shRNA. The expression of ULK1 was measured by Immunofluorescence staining, scale bar: 50 μm (**D**). Quantification of immunofluorescence staining images (**E**). (**F and G**) MDA-MB-231 xenograft specimens were subjected to IHC staining for ULK1, scale bar: 100 μm (**F**). Quantification of IHC staining images (**G**). (**H-J**) The expression of *NSD2* in a panel of 3 TNBC cell lines and normal MCF-10A cells were measured by qRT-PCR (**H**) and western blotting (**I**), Quantification of western blotting images (**J**). Error bars represented the mean ± SEM and the dots represented the value of each experiment. * *p* < 0.05, ** *p* < 0.01.


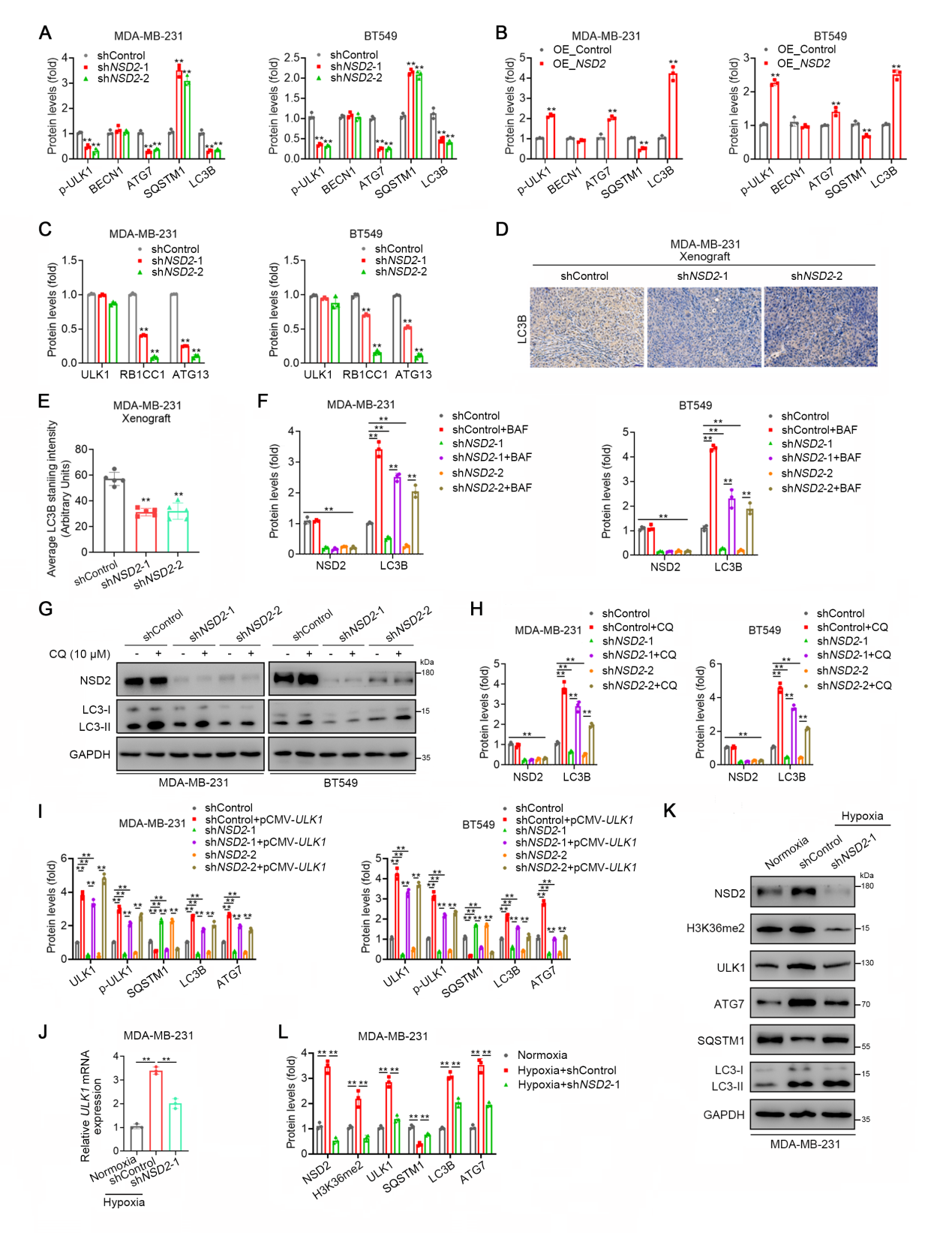


**Figure S5.** NSD2 drives ULK1-induced autophagy in TNBC. (**A**) Quantification of western blotting images in (Figure 5A). (**B**) Quantification of western blotting images in (Figure 5B). (**C**) Quantification of western blotting images in (Figure 5C). (**D and E**) MDA-MB-231 xenograft tumor specimens were subjected to IHC staining for LC3B, scale bar: 100 μm (**D**). Quantification of IHC staining images (**E**). (**F**) Quantification of western blotting images in (Figure 5F). (**G and H**) MDA-MB-231 and BT549 cells were treated with or without CQ (10 μM) for 24 h, and the expression of NSD2 and LC3B were measured by western blotting (**G**). Quantification of western blotting images (**H**). (**I**) Quantification of western blotting images in (Figure 5M). (**J-L**) *NSD2*-knockdown MDA-MB-231 cells and control cells were cultured under normoxia (20% O_2_) or hypoxia (1% O_2_). mRNA levels of *ULK1* were measured by qRT-PCR (**J**). Protein levels of NSD2, H3K36me2, ULK1, ATG7, SQSTM1, and LC3B were measured by western blotting (**K**). Quantification of western blotting images (**L**). Error bars represented the mean ± SEM and the dots represented the value of each experiment. * *p* < 0.05, ** *p* < 0.01.


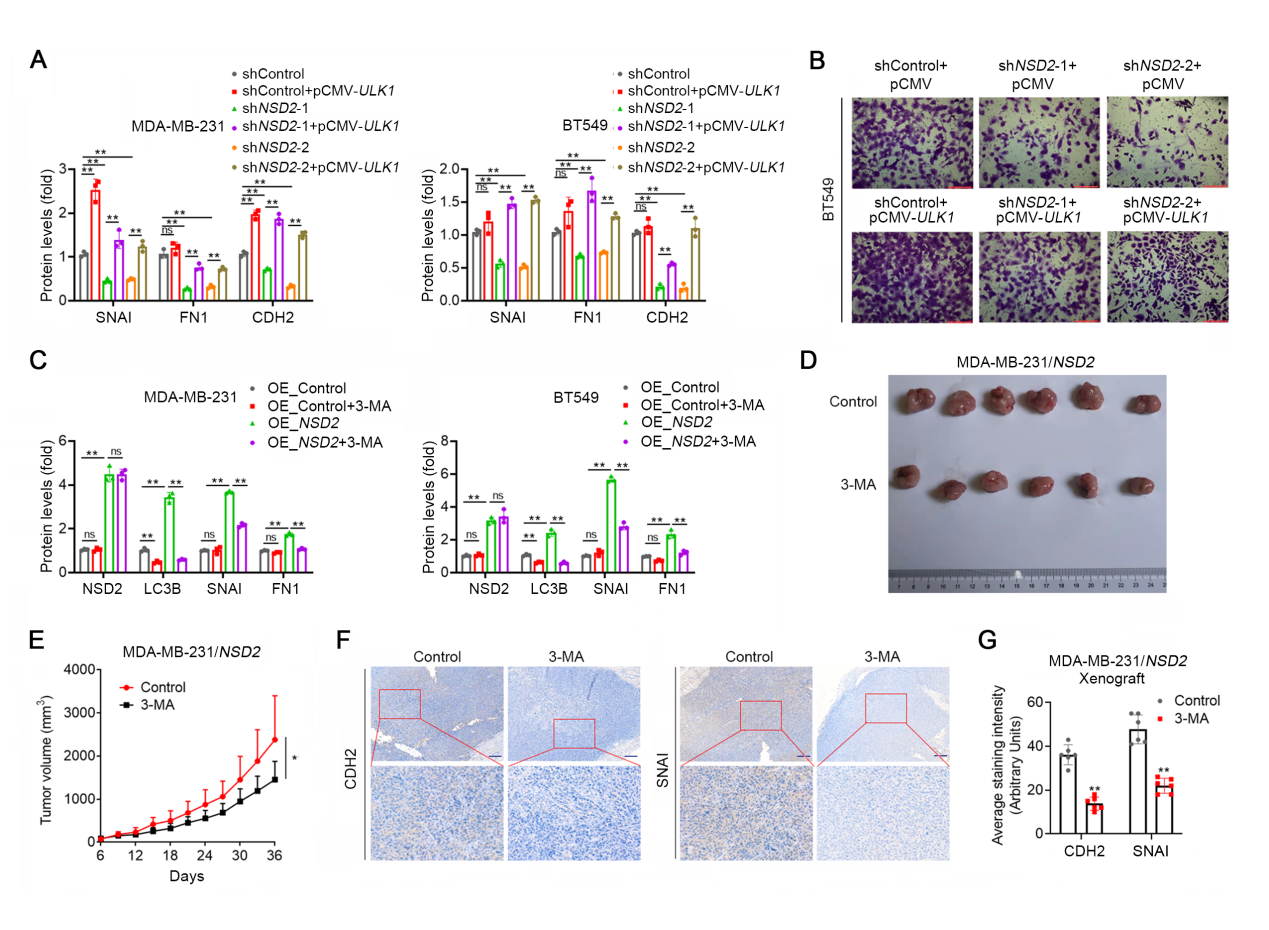


**Figure S6.** NSD2 promotes autophagy-associated TNBC progression. (**A**) Quantification of western blotting images in (Figure 7A). (**B**) *NSD2*-knockdown BT549 cells were transfected with pCMV-*ULK1* ORF vector or control vector, cell invasion ability was measured by transwell assay. Scale bar: 100 μm (**C**). Quantification of western blotting images in (Figure 7D). (**D and E**) 5 × 10^6^ MDA-MB-231/*NSD2* cells were inoculated subcutaneously into female nude mice and palpable tumors were allowed to develop for 6 days, mice were randomly allocated into two groups (N = 6 per group): vehicle control (Water) and 3-MA (30 mg/kg/day) via intraperitoneal (i.p.) injection for 36 days. At the end of treatment, tumors were excised and imaged (**D**). Tumor size was measured at indicated time intervals and tumor volume was calculated (**E**). (**F and G**) Tumor specimens were subjected to IHC staining for CDH2 and SNAI, scale bar: 100 μm (**F**). Quantification of IHC staining images (**G**). Error bars represented the mean ± SEM and the dots represented the value of each experiment. * *p* < 0.05, ** *p* < 0.01.


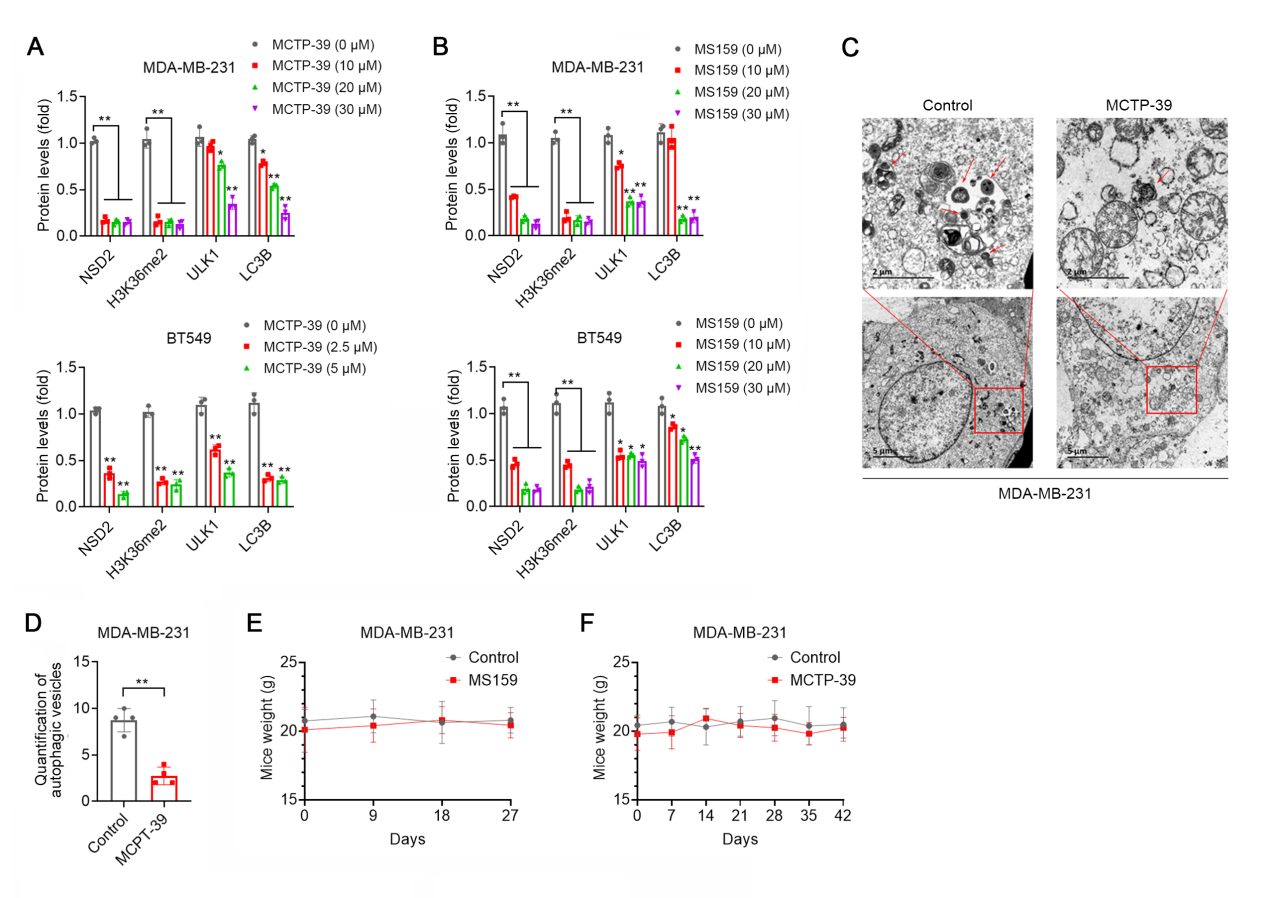


**Figure S7.** Targeting NSD2 inhibits TNBC autophagy, growth, and metastasis. (**A**) Quantification of western blotting images in (Figure 7A). (**B**) Quantification of western blotting images in (Figure 7B). (**C and D**) MDA-MB-231 cells were treated with MCTP-39 for 48 h. Transmission electron microscopy (TEM) demonstrating autolysosomes and autophagosomes in MDA-MB-231 cells (**C**). (**D**) Quantification of autophagic vesicles. (**E and F**) The body weight of experimental mice during the treatment was shown. Error bars represented the mean ± SEM and the dots represented the value of each experiment. * *p* < 0.05, ** *p* < 0.01.

**Table S1.** Primer sequences of qRT-PCR and ChIP-qPCR used in this study.

| Primer | Sequence |  |
| --- | --- | --- |
| GAPDH | Forward: 5′-GTCTCCTCTGACTTCAACAGCG-3′ | For qRT-PCR |
|  | Reverse: 5′-ACCACCCTGTTGCTGTAGCCAA-3′ |  |
| NSD2 | Forward: 5′-TGTGTGAGCTGCCATGCTTCCA-3′ | For qRT-PCR |
|  | Reverse: 5′-TGAGCATCCTGCTGCCAGACAA-3′ |  |
| ULK1 | Forward: 5′-GCAAGGACTCTTCCTGTGACAC-3′ | For qRT-PCR |
|  | Reverse: 5′-CCACTGCACATCAGGCTGTCTG-3′ |  |
| ULK1 #1 | Forward: 5′-AATCCCATCTTGCCATCC-3′ | For ChIP-qPCR |
|  | Reverse: 5′-GCGAAGCCTGTACTCCTG-3′ |  |
| ULK1 #2 | Forward: 5′-GCCACGGGTGGTGCAATCT-3′ | For ChIP-qPCR |
|  | Reverse: 5′-ACACGCTGCGAGGTAAGT-3′ |  |
| ULK1 #3 | Forward: 5'-AATGCTATGGCTGACTACGG-3' | For ChIP-qPCR |
|  | Reverse: 5'-CGAGCTGGAAGGGACAAG-3' |  |
| ULK1 #4 | Forward: 5'-GCAATCGCTGTTGGCTGTG-3' | For ChIP-qPCR |
|  | Reverse: 5'-CGAGGCACTTGCTGTTATGT-3' |  |
| ULK1 #5 | Forward: 5'-TGCTTGGCCTAAGTGATG-3' | For ChIP–qPCR |
|  | Reverse: 5'-AGAAAGCGGTAGGATTGG-3' |  |
| ULK1 #6 | Forward: 5'-TTATGGGCTATGTCGTTG-3' | For ChIP-qPCR |
|  | Reverse: 5'-GAGTTAGAGGTTAGGCATTT-3' |  |
